# MrgprF acts as a tumor suppressor in cutaneous melanoma by restraining PI3K/Akt signaling

**DOI:** 10.1038/s41392-022-00945-9

**Published:** 2022-05-04

**Authors:** Qiushuo Shen, Yanfei Han, Kai Wu, Yaomei He, Xiulin Jiang, Peishen Liu, Cuifeng Xia, Qiuxia Xiong, Rui Liu, Qianming Chen, Yong Zhang, Song Zhao, Cuiping Yang, Yongbin Chen

**Affiliations:** 1 Department of Thoracic Surgery, the First Affiliated Hospital of Zhengzhou University, Zhengzhou, 450052 China; 2 Key Laboratory of Animal Models and Human Disease Mechanisms of Chinese Academy of Sciences & Yunnan Province, Kunming Institute of Zoology, Kunming, Yunnan 650223 China; 3 Kunming Medical University, Kunming, Yunnan 650118 China; 4 State Key Laboratory of Oral Diseases, National Clinical Research Center for Oral Diseases, Chinese Academy of Medical Sciences Research Unit of Oral Carcinogenesis and Management, West China Hospital of Stomatology, Sichuan University, Chengdu, Sichuan China; 5 Department of Pathology, Cancer Hospital of China Medical University, Shenyang, Liaoning 110042 China; 6 The International Peace Maternity and Child Health Hospital, School of Medicine, Shanghai Jiao Tong University, Shanghai, 200030 China; 7 Shanghai Key Laboratory of Embryo Original Diseases, Shanghai, 200030 China

**Keywords:** Skin cancer, Molecular medicine

## Abstract

The incidence of cutaneous melanoma (CM) has been increasing annually worldwide. In this study, we identify that MrgprF, a MAS related GPR family member, is decreased in cutaneous melanoma tissues and cell lines due to hypermethylation of its promoter region, and show that patients with CM expressing high levels of MrgprF exhibit an improved clinical outcome. We demonstrate that MrgprF forced expression inhibits tumor cell proliferation, migration, xenograft tumor growth, and metastasis. On the contrary, MrgprF knockdown promotes tumor cell proliferation and transformation of immortalized human keratinocyte-HaCaT cells, supporting the inhibitory role of MrgprF during tumor progression. Mechanistic studies reveal that MrgprF reduces the phosphoinositol‑3‑kinase (PI3K) complex formation between p101 and p110γ subunits, the critical step for phosphatidylinositol-(3, 4)-P2 (PIP2) conversion to phosphatidylinositol-(3, 4, 5)-P3 (PIP3), and then reduces the activation of PI3K/Akt signaling. This effect can be reversed by Akt specific agonist SC79. In addition, AMG 706, a previously documented inhibitor for endothelial cell proliferation, is identified as a potential agonist for MrgprF, and can impede tumor growth both in vitro and in vivo. Taken together, our findings suggest that MrgprF, a novel tumor suppressor in cutaneous melanoma, may be useful as a therapeutic target in the future.

## Introduction

The incidence of cutaneous melanoma (CM), the leading cause of death from skin cancer, has increased rapidly worldwide in recent years compared to other types of human cancer.^1,2^ Advanced age and Caucasian race are the two critical risk factors for CM according to US epidemiologic data,^1^ and indoor tanning (especially in young women) has also been indicated as a risk factor.^3^ S100 calcium-binding protein-p, MLANA (Melanoma-Antigen recognized by T-cells 1), and MITF (microphthalmia-associated transcription factor) are regularly used as markers for ambiguous cases showing low specificity.^4^ The identification of genetic mutations in BRAF (B‑Raf proto‑oncogene), CDKN2a (cyclin-dependent kinase inhibitor 2 A) and NRAS (neuroblastoma RAS viral oncogene homolog) have been described to increase the accuracy of diagnosis.^5^ Previous studies have demonstrated that CM progression results from both genetic mutations and tumor microenvironmental alterations, as evidenced by the activation of signaling pathways favoring tumor invasion and infiltration. For example, matrix metalloproteinases (MMPs), including MMP-2 and MMP-9, have been demonstrated to be induced by the NF-κB signaling pathway in melanoma.^6,7^

Apart from tumor microenvironment alterations, the somatic mutations in BRAF, CDKN2a, NRAS, NF1 (Neurofibromatosis type 1), TP53, PTEN, and TERT (The telomerase reverse transcriptase), mainly lead to intrinsic activation of the mitogen‑activated protein kinase (MAPK) pathway and the phosphoinositol‑3‑kinase (PI3K)/Akt pathway.^8,9^ The MAPK pathway involved in the transduction of growth factors and hormones has been shown to be activated in different types of cancer.^10,11^ The PI3K/Akt pathway is routinely activated by receptor tyrosine kinases (RTKs) and G protein-coupled receptors (GRCRs), leading to the increased conversion of phosphatidylinositol-(3,4)-P2 (PIP2) to phosphatidylinositol-(3,4,5)-P3 (PIP3), as well as high level of phosphorylation on Akt proteins.^12^ However, there is still much work remaining to be done to improve the earlier detection of deadly CMs, due to lack of reliable diagnostic biomarkers.

Most patients with CM should undergo dermatologic examination annually, and surgical excision is still the first choice for the majority of curative cases, while patients at a more advanced stage may be unresectable or already metastatic.^13,14^ In recent years, melanoma treatment has been revolutionized by applying targeted and/or immuno-therapies, including BRAF and MEK (MAP-ERK kinase) inhibitors, and immune checkpoint inhibitors (anti-cytotoxic T-lymphocyte- associated antigen 4 antibodies and anti‑programmed cell death protein 1 antibodies).^8^

Our recent study identified multiple pan-cancer biomarkers which are unanimously deregulated in various types of human cancer.^15^ By using integrative bioinformatic analysis, in combination with other web-source available CM related datasets, we identified that *MRGPRF*, a MAS related GPR family member, is decreased in multiple types of human cancer, including cutaneous melanoma. Mas related genes (*Mrgs*) belong to the GPCR family of genes, which are predominantly expressed in subsets of sensory neurons in the dorsal root ganglia (DRGs) and trigeminal ganglia (TG), with most studies focusing on itch and pain sensation.^16,17^ However, the deregulation of *Mrgs* outside the nervous system has not been thoroughly investigated, with only a few references. For example, MrgprD, was shown to be upregulated in non-small cell lung cancer and the mouse intestinal tract.^18–20^ *MRGX2* has been shown to participate in cancer progression after activation by LL-37, a group of antimicrobial peptide gene family cathelicidins.^21^ These findings indicate that *Mrgs* play pivotal roles in other normal human developmental processes and pathological conditions, such as human cancers. Therefore, we decided to uncover the functional roles of MrgprF in CM by determining its molecular mechanism, and screen for potential anti-cancer compounds targeting MrgprF. These compounds could provide therapeutic drugs for CM treatment in the future.

## Results

### MrgprF is decreased in cutaneous melanoma

**Fig. 1:**
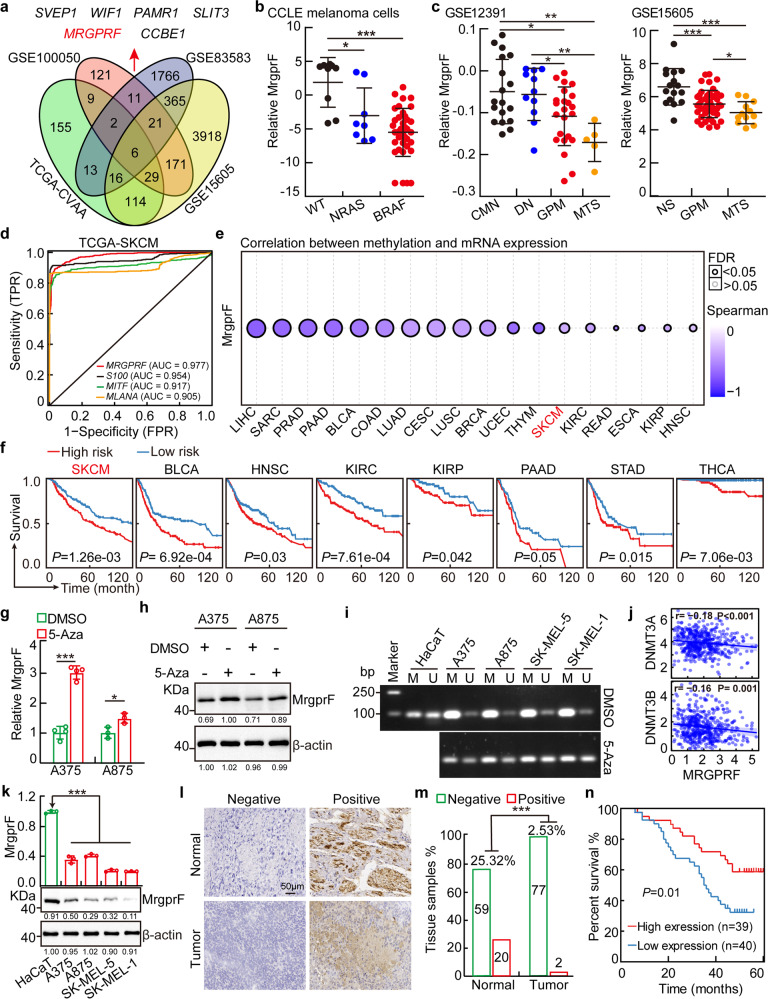
MrgprF was decreased in melanoma. **a** MrgprF was identified by integrative bioinformatics analysis using TCGA-CVAA (pan-cancer deregulated genes, green) dataset and melanoma related GEO datasets, including GSE100050 (red), GSE83583 (blue), and GSE15605 (yellow). Six shared DEGs, including *SVEP1*, *WIF1*, *PAMR1*, *SLIT3*, *MRGPRF*, and *CCBE1* were identified. **b** MrgprF was decreased in NRAS and BRAF mutated cell lines examined by the CCLE (the cancer cell line encyclopedia) dataset, NRAS and BRAF wild type cells were used as control. **c** MrgprF is downregulated in advanced melanoma compared to normal nevi or primary melanoma as analyzed by GSE12391 and GSE15605 datasets. CMN: common melanocytic nevi; DN dysplastic nevi; GPM growth phase melanomas; MTS melanoma metastasis; NS normal skin. **d** Receiver operating characteristic (ROC) curves for MrgprF, S100, MITF, and MLANA examined by TCGA-melanoma datasets. The AUC (area under the curve) numbers are indicated. **e** MrgprF mRNA expression level was negatively correlated with its promoter methylation status in melanoma analyzed by the GSCALite database. **f** High methylation levels in the MrgprF promoter region (high risk) positively correlated with poor survival probability in various types of human cancer, as analyzed by the DNMIVD database in comparison to low risk group. **g**, **h** MrgprF expression was examined by Real-time RT-PCR (**g**) and immunoblot (**h**) after 5-Azacytidine (5-Aza) treatment. **i** Methylation-specific PCR (MSP) experiment validating the methylation status of *MRGPRF* promoter with or without 5-Aza treatment. M: methylated alleles; U: unmethylated alleles. **j** Correlation analysis between MrgprF and DNMT3A/DNMT3B, using the TCGA-melanoma database. **k** The relative expression levels of MrgprF in melanoma cell lines including A375, A875, SK-MEL-5, and SK-MEL-1 examined by Real-time RT-PCR and immunoblot. The human immortalized keratinocyte-HaCaT cell line was used as a control. **l**, **m** Representative immunohistochemistry (IHC) staining images for MrgprF using a melanoma tissue microarray (TMA) (**l**). The statistical data for IHC staining was also indicated (**m**). Scale bar = 50 μm. **n** MrgprF low expression positively correlates with poor survival rate analyzed by TMA dataset in (**l**, **m**). Quantified results for all the immunoblots are indicated below, which are normalized to the β-actin signal, compared to reciprocal control. Bars are the mean value ± SD. *P < 0.05, **P < 0.01, ***P < 0.001

To identify novel biomarkers in cutaneous melanoma, we used an integrative bioinformatics analysis, combining our previous identified pan-cancer deregulated genes (DEGs) with web-source available datasets in CM.^15^ Six shared DEGs were identified, including *SVEP1*, *WIF1*, *PAMR1*, *SLIT3*, *MRGPRF* and *CCBE1* (Fig. 1a, Supplementary Table S1). Among them, *SVEP1*, *WIF1*, *PAMR1*, *SLIT3*, and *CCBE1* have been thoroughly studied in various types of human cancer, including CM.^22–26^ Therefore, we decided to further explore the functional role of MrgprF. First, we examined the expression profile of MrgprF in human tissues using RNA sequencing data retrieved from the GTEX database (http://www.gtexportal.org), and showed that MrgprF is ubiquitously expressed in most human tissues, including skin (Supplementary Fig. S1a, Supplementary Table S2). Consistently, the expression pattern of MrgprF in mouse vital tissues was also shown, with higher expression in skin and lung tissues (Supplementary Fig. S1b). We then examined the expression profile of MrgprF in various types of human cancer using GEPIA and CCLE (the cancer cell line encyclopedia) datasets.^27,28^ Consistent with our previous result,^15^ MrgprF was found to be unanimously downregulated in multiple human cancerous tissues and cell lines, including cutaneous melanoma, compared to reciprocal controls (Supplementary Fig. S1c, d, Supplementary Table S3–4). Furthermore, we revealed that MrgprF low expression positively correlates with RAS and BRAF mutations in the CCLE melanoma dataset (Fig. 1b). We also found that MrgprF is markedly decreased in advanced stage melanoma, compared to common melanocytic nevi (CMN) and primary melanoma, by analyzing whole genome oligo-microarray datasets (Fig. 1c). In addition, the mutation profile of MrgprF was uncovered using the cBioPortal web-source^29^ and GSCALite database (Supplementary Fig. S1e, Supplementary Table S3–5).^30^ CM patients with *MRGPRF* alteration exhibit a trend toward inferior overall survival rate compared to a reciprocal control group (Supplementary Fig. S1f). The ROC curve indicated that MrgprF could be used as an independent prognostic biomarker in melanoma (AUC = 0.977), compared to other documented biomarkers, including S100 (AUC = 0.954), MITF (AUC = 0.917) and MLANA (AUC = 0.905) (Fig. 1d).^4^

To decipher the mechanism by which MrgprF is downregulated in various tumors, the correlation between the MrgprF promoter methylation and expression level were examined using multiple web-source datasets, including GSCALite, DNMIVD, and MethSurv.^30–32^ Hypermethylation in the MrgprF promoter region was shown to be negatively associated with its transcript expression level and the overall survival rate in multiple tumors, including CM (Fig. 1e, f, Supplementary Fig. S1g). In addition, we showed that MrgprF decreased expression in CM could be reversed by 5-Azacytidine (5-Aza) treatment, a specific inhibitor for DNA methylases (Fig. 1g, h).^33^ Similarly, the methylation-specific PCR (MSP) and results confirmed the hypermethylation of the MrgprF promoter in CM cells compared to that in HaCaT cells, which was erased by 5-Azacytidine treatment (Fig. 1i). The negative correlation between *MRGPRF* and the DNA methyltransferase (DNMT) family members, including DNMT3A and DNMT3B, but not DNMT1, was also detected (Fig. 1j).^34^ We revealed that knockdown of DNMT3A or DNMT3B, respectively, increased MrgprF expression, indicating that the hypermethylation in the MrgprF promoter region depends on both DNMT3A and DNMT3B (Supplementary Fig. S1i–k).

To validate the downregulation of MrgprF in CM, both the mRNA and protein expressions of MrgprF in cancerous cell lines (A875, A375, SK-MEL-1, and SK-MEL-5) and immortalized human keratinocyte-HaCaT cells was examined, and MrgprF was revealed to be markedly decreased in tumor cells (Fig. 1k). Furthermore, the DNA methylation sequencing result confirmed that the methylation level is higher in MrgprF promoter region in A375 cells compared to that in HaCaT control cells, which could be reversed by 5-Azacytidine treatment (Supplementary Fig. S1h). Consistently, the decreased expression of MrgprF was detected by immunohistochemistry (IHC) staining using CM cancerous tissue microarrays (Fig. 1l, m). Furthermore, CM patients with lower MrgprF expression exhibited worse overall survival time compared to those expressing higher MrgprF (Fig. 1n, Supplementary Table S6). These results prompted us to hypothesize that decreased expression of MrgprF is critical for tumorigenesis in CM.

### MrgprF inhibits tumor growth both in vitro and in vivo

**Fig. 2:**
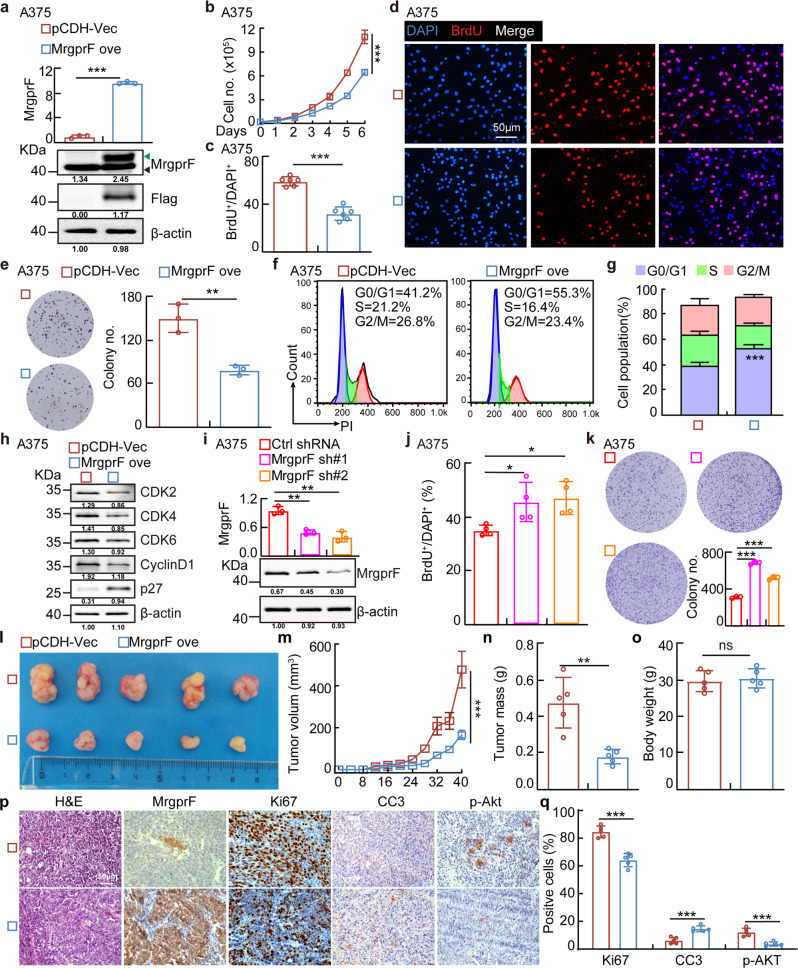
MrgprF overexpression inhibited tumor cell proliferation. **a** Establishment of MrgprF overexpression in A375 cells, verified by Real-time RT-PCR (top) and immunoblot (bottom). Green arrow: exogenous MrgprF-Flag; black arrow: Endogenous MrgprF. **b** Indicated cell growth was examined by daily counting. **c**, **d** MrgprF overexpression reduced BrdU positive staining cells by BrdU incorporation assay in A375 cells. **c** Quantification data for (**d**). Scale bar = 50 μm. **e** MrgprF overexpression impeded the colony formation ability of A375 cells. Quantification data was also shown (right). **f**, **g** MrgprF forced expression caused G0/G1 phase arrested cell population as examined by FACS analysis. **g** Quantification data for (**f**). **h** Overexpression of MrgprF decreased the expressions of CDK2, CDK4, CDK6, Cyclin D1, but increased p27 expression. Indicated cell extracts were probed with indicated antibodies. **i** Establishment of MrgprF knockdown cell lines in A375 cells, verified by Real-time RT-PCR (top) and immunoblot (bottom), respectively. **j** BrdU incorporation assays were performed in A375 cells. **k** MrgprF knockdown promoted colony formation abilities of A375 cells. Quantification data is also shown. **l**–**o** MrgprF overexpression markedly repressed xenograft tumor growth. Representative xenograft tumor images (**l**), and quantification data for tumor volumes (**m**, data are presented as mean ± SEM), tumor masses (**n**) and mice body weights (**o**) are shown. **p**, **q** Representative IHC staining images of Ki67, Cleaved Caspase 3 (CC3) and phosphorylated form of Akt (p-Akt) for indicated xenograft tumors (**p**), and the quantification results are also indicated (**q**). Scale bar = 50 μm. Quantified results for all the immunoblots are indicated below, which are normalized to the β-actin signal, compared to reciprocal control. Bars are the mean value ± SD. *P < 0.05, **P < 0.01, ***P < 0.001. Colony no. Colony numbers; Ctrl Scramble control shRNA; sh#1 shRNA#1; sh#2 shRNA#2; pCDH-vec empty vector control; MrgprF ove MrgprF overexpression; HPF High power field

The gene set enrichment analysis (GSEA) showed that MrgprF is significantly associated with the cell cycle related signaling pathway (Supplementary Fig. S2a).^35^ To investigate the functional role of MrgprF in CM, we overexpressed a C-terminal Flag-tagged MrgprF protein using a lenti-viral system. Both the overexpressed MrgprF mRNA and protein were verified by Real-time RT-PCR and immunoblot assays (Fig. 2a, Supplementary Fig. S2b). We showed that overexpression of MrgprF inhibited tumor cell growth compared to the control cells. Decreased BrdU-positive cell population and cell colonies were also detected in A375 and A875 cells upon MrgprF forced expression (Fig. 2b–e, Supplementary Fig. S2c–g). To validate whether MrgprF is critical for cell cycle transition, we performed flow cytometry analysis which revealed that MrgprF forced expression led to increased G0/G1 phase arrested cells (Fig. 2f, g, Supplementary Fig. S2h, i). Furthermore, more p27 proteins, but less CDK2, CDK4, CKD6, and Cyclin D1 proteins in MrgprF overexpressing cells were detected compared to that in control cells (Fig. 2h, Supplementary Fig. S2j). On the contrary, we showed that MrgprF knockdown by two independent targeting shRNAs, in A375 or HaCaT cells, promoted cell growth and colony formation (Fig. 2i–k, Supplementary Fig. S2k–m). To verify whether the cell cycle transition blockage caused by MrgprF overexperssion led to cellular apoptosis, apoptosis with or without the chemotherapy drug cisplatinum (DDP) or BRAF-targeted therapy drug, Vemurafenib,^36^ treatment was examined. Although the baseline cellular apoptosis was not affected upon MrgprF forced expression, MrgprF overexpression induced more apoptotic cells compared to the control group with, but not without, DDP or Vemurafenib treatment, respectively (Supplementary Fig. S2n–p).

To determine the function of MrgprF in vivo, we performed a xenograft tumor formation assay. Male nude mice were randomly divided into two groups, MrgprF overexpressing and control cell lines were subcutaneously injected (1 × 10^6^ cells/point), and the tumor growth was measured every 4 days. As expected, we observed subcutaneous tumors in the vector control group, while the MrgprF overexpression group robustly retarded tumor growth in vivo, with decreased tumor weights and volumes compared to control group (Fig. 2l–o). Consistently, a lower proliferation rate evidenced by less Ki67 positive staining, and an increase of apoptosis evidenced by increased CC3 positive staining, were detected in the xenograft tumors from the MrgprF overexpressing group compared to the control group, supporting the hypothesis that MrgprF forced expression impedes melanogenesis (Fig. 2p, q).

### MrgprF acts as a tumor suppressor inhibiting tumor metastasis

**Fig. 3:**
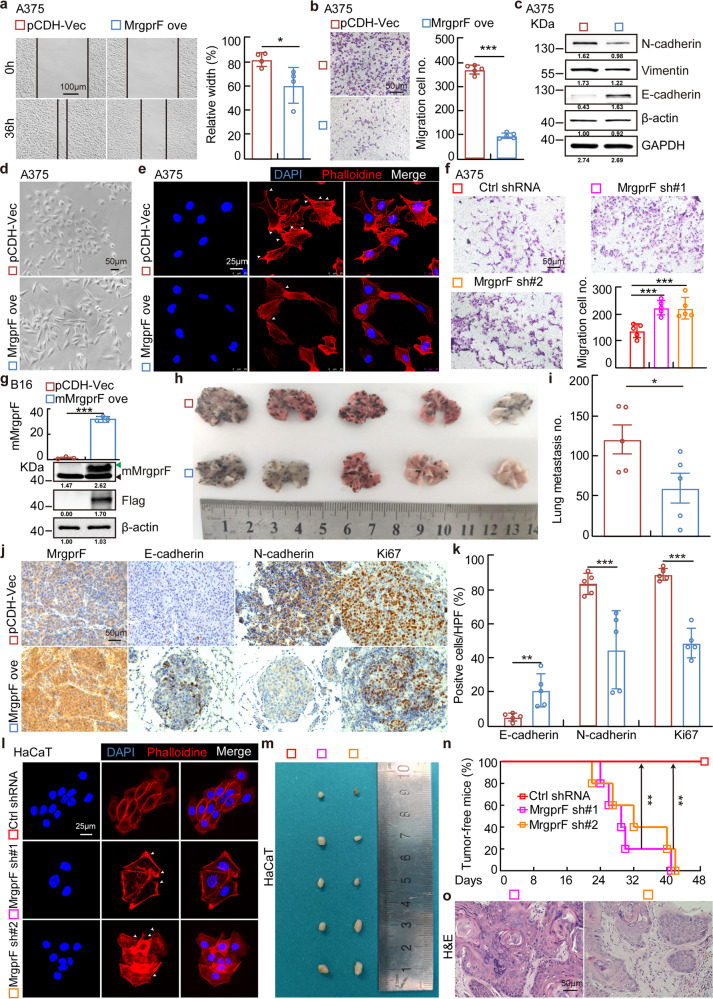
MrgprF overexpression inhibited tumor cell migration. **a**, **b** MrgprF overexpression inhibited A375 cell migration by wound healing (**a**) and trans-well assays (**b**) in indicated A375 cells. Quantification data were also indicated for each assay. **c** Indicated cell extracts were probed with indicated antibodies to examine the protein expressions of E-cadherin, N-cadherin, and Vimentin. **d** Representative images showing the morphological change in A375 after MrgprF overexpression under bright field. Scale bar = 50 μm. **e** Immunofluorescence staining of Phalloidine in indicated cells. White head arrows pointing to the pseudopodia like structure. Scale bar = 25 μm. **f** MrgprF knockdown in A375 promoted cell migration ability as determined by trans-well assay. Quantification data is also shown. Scale bar = 50 μm. **g** Establishment of mouse MrgprF (mMrgprF) overexpression in mouse B16 cells, verified by Real-time RT-PCR (top) and immunoblot (bottom). Green arrow: exogenous mMrgprF-Flag; black arrow: endogenous mMrgprF. **h**, **i** Lung metastasis model using B16 cells in C57BL/6 mice. Representative images for the lung tissues (**h**) and the quantification result for the metastatic tumor numbers (**i**) are shown. **j**, **k** Representative IHC staining images for MrgprF, E-cadherin, N-cadherin and Ki67. **k** Quantification data for (**j**). **l** Immunofluorescence staining of phalloidine in HaCaT cells after MrgprF knockdown. White head arrows pointing to the pseudopodia like structure. Scale bar = 25 μm. **m**–**o** HaCaT cells stably expressing control or *MRGPRF* targeting shRNAs were subcutaneously injected (2 × 10^7^ cells/mouse) into nude mice, and representative xenograft tumor images (**m**), tumor-free mice percentage (**n**) and H&E staining images of the xenograft tumors (**o**) are shown. Scale bar = 50 μm. Quantified results for all the immunoblots are indicated below, which are normalized to the β-actin signal, compared to reciprocal control. Bars are the mean value ± SD. *P < 0.05, **P < 0.01, ***P < 0.001

To examine the potential role of MrgprF in regulating tumor cell migration, we performed wound healing and trans-well assays. In line with the bioinformatic analysis results, showing that MrgprF is associated with cell migration related signaling pathways (Supplementary Fig. S3a), we found that forced expression of MrgprF inhibits cell migration by inducing E-cadherin, but reducing N-cadherin and Vimentin expression, in A875 and A375 cells, compared to the control group (Fig. 3a–c, Supplementary Fig. S3b, d). Consistently, the cell morphological change examined by phalloidin immunofluorescence staining validated the mesenchymal-epithelial transition status in MrgprF overexpressing cells (Fig. 3d, e). On the contrary, we showed that MrgprF knockdown promoted A375 and HaCaT cell migration (Fig. 3f, Supplementary Fig. S3e). Based on the functional role of MrgprF in regulating tumor cell migration in vitro, we used a lung metastasis mouse model to assess the effect of MrgprF overexpression in vivo. We found that forced expression of highly conserved mouse MrgprF (mMrgprF) in B16, a mouse melanoma cell line, significantly inhibited the formation of lung metastasis, visualized by a dramatic decrease in tumor number compared to the control group (Fig. 3g–i, Supplementary Fig. S3f). As expected, the expressions of Ki67 and N-cadherin were markedly decreased, while MrgprF and E-cadherin were increased compared to B16 cells expressing a control vector (Fig. 3j, k). Importantly, we have provided evidence showing that MrgprF knockdown in HaCaT cells promotes their metastatic transformation and xenograft tumor growth (Fig. 3l–o). The above data indicate that MrgprF acts as a novel tumor suppressor in cutaneous melanoma.

### MrgprF restrains PI3K/Akt signaling

**Fig. 4:**
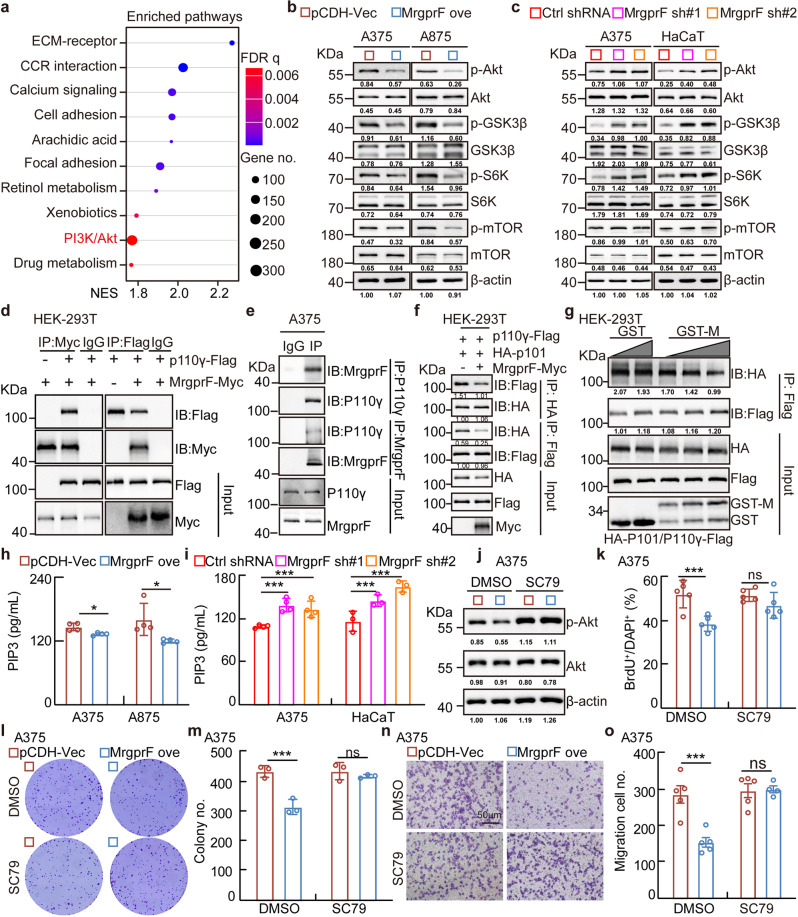
MrgprF inhibited PI3K/Akt signaling activation. **a** MrgprF was involved in PI3K/Akt signaling pathway by Kyoto Encyclopedia of Genes and Genomes (KEGG) pathway analysis using LinkedOmics portal datasets. **b**, **c** MrgprF overexpression decreased (**b**), while its knockdown promoted (**c**), the phosphorylation levels of the major mediators in the PI3K/Akt signaling pathway, including Akt, GSK3β, S6K, and mTOR, without affecting the total protein expressions in A375 and A875. **d**, **e** Co-immunoprecipitation assay (co-IP) determining the exogenous (**d**) and endogenous (**e**) protein interaction between MrgprF and p110γ. IP: immunoprecipitation, IB: immunoblot. **f** MrgprF overexpression reduced the protein-protein interaction between p110γ and p101 as examined by co-IP assay. Indicated constructs were transfected into HEK-293T cells and cell lysates were used for co-IP and IB with the indicated antibodies. **g** GST-MrgprF_274-373_ (100 nM, 200 nM, 300 nM), but not GST proteins (100 nM, 200 nM), markedly reduced the interaction between exogenous p110γ-Flag and HA-p101. HEK-293T cells were transfected with p110γ-Flag and HA-p101 constructs, and the cell extracts were randomly divided for indicated competition co-IP assay. GST-M = GST-MrgprF_274-373_. **h**, **i** The quantification data for PIP3 products upon MrgprF overexpression (**h**) or knockdown (**i**) in indicated cells, examined by ELISA. **j** Indicated cells stably expressing empty vector or MrgprF in A375 cells were treated with DMSO or SC79 (15 μM). Indicated cell lysates were examined by immunoblot with the indicated antibodies. **k** The quantification data for the BrdU incorporation assay in A375 upon MrgprF overexpression treated with DMSO or SC79 (15 μM). **l**, **m** The colony formation ability of A375 after MrgprF forced expression treated with DMSO or SC79 (15 μM). **m** Quantification data for (**l**). **n**, **o** The trans-well assay for A375 upon MrgprF overexpression treated with DMSO or SC79 (15 μM). **o** Quantification data for (**n**). Scale bar = 50 μm. Quantified results for all the immunoblots are indicated below, which are normalized to the β-actin signal, compared to reciprocal control. Bars are the mean value ± SD. *P < 0.05, **P < 0.01, ***P < 0.001

To decipher the underlying mechanism, Kyoto Encyclopedia of Genes and Genomes (KEGG) pathway analysis showed that MrgprF is involved in PI3K/Akt, but not the MAPK signaling pathway (Fig. 4a, Supplementary Fig. S4a, b). Immunoblot results confirmed that MrgprF overexpression inhibited, while MrgprF knockdown promoted, the phosphorylation of PI3K/Akt signaling downstream effectors, including Akt, GSK3β, mTOR, and S6K, normalized to reciprocal total proteins (Fig. 4b, c). Based on the fact that Class 1 A (heterodimeric enzymes composed of a regulatory subunit p85 and catalytic subunit p110α/β/δ) and 1B (heterodimeric enzymes composed of a regulatory subunit p101 and catalytic subunit p110γ) PI3Ks are critical for the signal transduction of RTKs and GPCRs,^12^ we sought to examine the protein-protein interactions between MrgprF and PI3Ks using a co-immunoprecipitation (co-IP) assay. We found that exogenous Myc-tagged MrgprF tightly bound to Flag-tagged p110γ, but not Flag-tagged p85, suggesting that the Class 1B PI3K complex might be essential for mediating the MrgprF inhibitory role in CM (Fig. 4d, Supplementary Fig. S4c). The endogenous MrgprF and p110γ interaction were confirmed in CM and other types of tumor cells (Fig. 4e, Supplementary Fig. S4d). To map the functional domain mediating the interaction between MrgprF and p110γ, we performed co-IP experiment using a series of MrgprF and p110γ mutants in HEK-293T cells based on previously defined protein domains,^37^ and identified that the MrgprF_295-343_ and p110γ_1-216_ fragments were critical to mediate the protein-protein interaction, which was further validated by GST-pull down assay (Supplementary Fig. S4e–h). In addition, the interaction between p101 and p110γ was markedly reduced by forced expression of MrgprF or adding exogenous purified GST-fused MrgprF_274-343_ protein, indicating that MrgprF competed with p101 to form a complex with p110γ, leading to reduced generation of PIP3 and downstream signaling activation (Fig. 4f–h). On the contrary, MrgprF knockdown resulted in increased PIP3 generation (Fig. 4i). As expected, the reduced Akt phosphorylation level and cell proliferation and migration abilities upon MrgprF overexpression were all reversed by SC79 treatment, a specific agonist for Akt,^38^ compared to a DMSO control treatment group (Fig. 4j–o, Supplementary Fig. S4i–n).

### AMG 706 activates MrgprF expression, while inhibits tumor growth

**Fig. 5:**
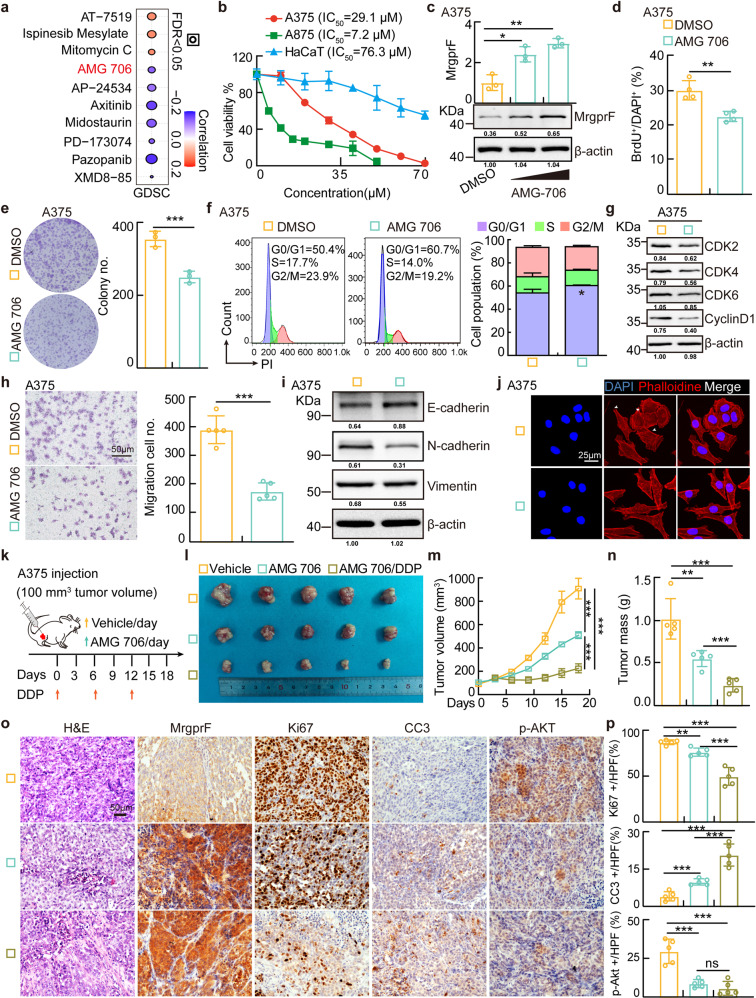
AMG 706 is a potent MrgprF agonist inhibiting CM progression. **a** Pearson correlation analysis was performed between MrgprF mRNA expression and drug IC_50_ using the GDSC database. Blue bubbles: negative correlations; Red bubbles: positive correlations, the deeper the color is, the higher the correlation is. Bubble size is positively associated with the FDR significance. Black outline border indicates FDR < 0.05. **b** Cell viabilities following treatment with the indicated concentration of AMG 706 for 48 h were examined by sulforhodamine B (SRB) staining. The IC_50_ for each cell line is indicated. **c** AMG 706 (15 and 30 μM) 24 h treatment increased MrgprF expressions examined by Real-time RT-PCR (top) and immunoblot (bottom) in A375 cells. **d**, **e** AMG 706 (15 μM) treatment in A375 cells decreased cell proliferation as examined by BrdU incorporation (**d**) and colony formation (**e**) assays. Quantification data are shown. **f**, **g** AMG 706 (15 μM) treatment in A375 cells promoted G0/G1 phase arrested cell population as examined by FACS (**f**) and immunoblot (**g**). Indicated cell lysates were probed with the indicated antibodies. Quantification data is shown. **h**, **i** AMG 706 (15 μM) treatment in A375 cells suppressed cell migration as examined by trans-well (**h**) and immunoblot (**i**). Indicated cell lysates were probed with indicated antibodies. Quantification data is shown. Scale bar = 50 μm. **j** Immunofluorescence staining of Phalloidine in A375 treated with DMSO or AMG 706 (15 μM). White head arrows pointing to the pseudopodia like structure. Scale bar = 25 μm. **k** Schematic view of xenograft mouse model treated by indicated drugs. **(l-n)** AMG 706 inhibited xenograft tumor growth in vivo with AMG 706 (7.5 mg/kg) or/and DDP (7 mg/kg) treatments. Representative xenograft tumor images (**l**), tumor volumes (**m**, data are presented as mean ± SEM) and tumor masses (**n**) are shown for the indicated mouse groups. A375 cells were used. **o**, **p** Representative IHC staining of MrgprF, Ki67, CC3, and p-Akt in indicated xenograft tumors. **p** Quantification data for (**o**). Scale bar = 50 μm. Quantified results for all the immunoblots are indicated below, which are normalized to the β-actin signal, compared to reciprocal control. Bars are the mean value ± SD. *P < 0.05, **P < 0.01, ***P < 0.001

To investigate the potential clinical value of MrgprF in CM, we used the GDSC database (http://bioinfo.life.hust.edu.cn/GSCA/#/expression) to predict the correlation between MrgprF expression and the sensitivity of GDSC available drugs in pan-cancers (Fig. 5a). Among the top candidate drugs, AMG 706, the well characterized inhibitor for VEGFRs,^39^ caught our attention because its IC_50_ negatively correlated with MrgprF expression, whose potential functional role in tumors, especially in CM, has never been defined (Fig. 5b, Supplementary Table S9). Indeed, we detected that AMG 706 preferentially inhibited the cell survival of A375 and A875 cells compared to HaCaT control cells. It also revealed that AMG 706 robustly increased both the mRNA and protein expressions of MrgprF in A375 and A875 cells by inhibiting DNMT3A/DNMT3B expression. (Fig. 5c, Supplementary Fig. S5a, b). Furthermore, we showed that AMG 706 inhibited the tumor cell proliferation, colony formation, and cell migration abilities, while promoted cellular apoptosis with or without DDP treatment, leading to reduced Akt phosphorylation and PI3K/Akt signaling activation (Fig. 5d–j, Supplementary Fig. S5c–h, S5j–l). In addition, we found that AMG 706 treatment reduced tumor cell proliferation and migration abilities could be reversed by MrgprF knockdown, possibly via reducing DNMT3A and DNMT3B expression (Supplementary Fig. S5m–o).

**Fig. 6:**
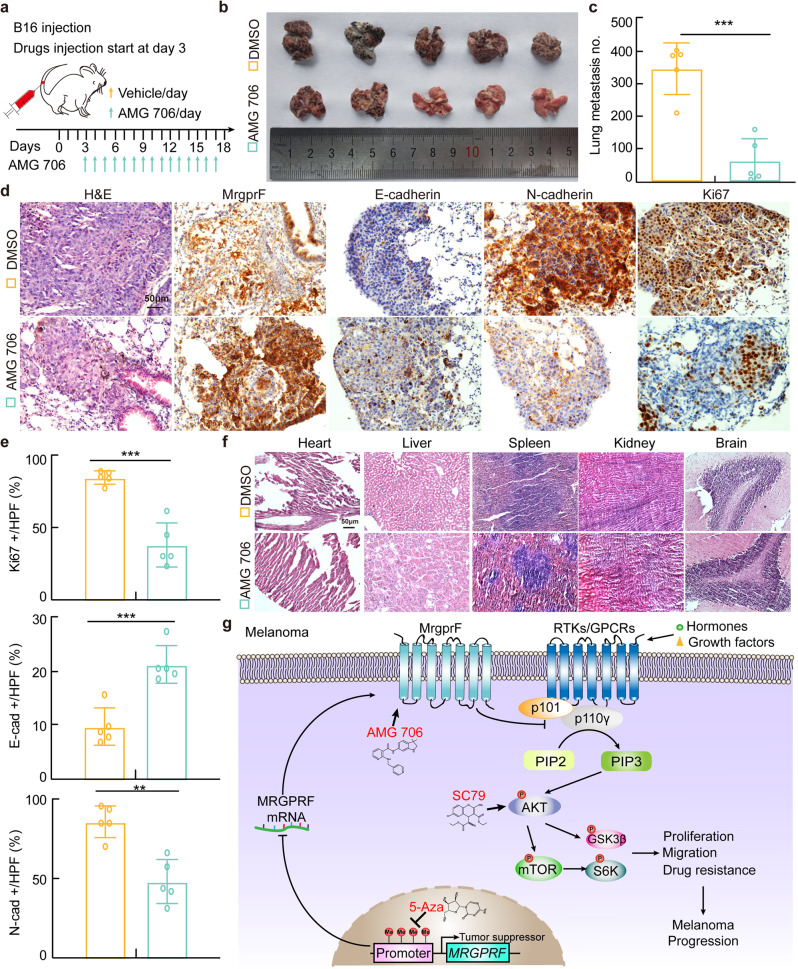
AMG 706 treatment inhibited the metastatic tumor progression. **a** Schematic view of the lung metastasis mouse model treated by indicated drugs. AMG706 and vehicle were injected daily starting from day 3 after B16 injection. **b**, **c** Representative lung tissues were shown (**b**), and the metastatic tumor numbers in lung tissues were quantified (**c**). **d**, **e** Representative images of the H&E and IHC staining using the indicated metastatic lung tumors. Antibodies for MrgprF, E-cadherin, N-cadherin, and Ki67 were used. **e** Quantification data for (**d**). Scale bar = 50 μm. E-cad: E-cadherin; N-cad: N-cadherin. **f** Representative images of the H&E staining of the vital organs in mice (Heart, Liver, Spleen, Kidney, and Brain), with or without AMG 706 treatment. **g** A model deciphering how MrgprF inhibited melanoma progression. Bars are the mean value ± SD. *P < 0.05, **P < 0.01, ***P < 0.001

The therapeutic effect of AMG 706 was also validated in vivo by subcutaneous xenograft tumor formation assay. Male nude mice were randomly divided into three groups, A375 cells (1 × 10^6^/point) were injected subcutaneously. Until the tumor reached about 100 mm^3^ volume, the mice were intraperitoneally injected with vehicle and AMG 706 (7.5 mg/kg) with or without DDP treatment (7 mg/kg) (Fig. 5k). The tumor masses and volumes were monitored. AMG 706 was revealed to markedly impede xenograft tumor growth compared with vehicle control treatment group, which can be synergized by DDP treatment as evidenced by the more robust reduction of xenograft tumor volumes and masses (Fig. 5l–n, Supplementary Fig. S5i). IHC staining showed that AMG 706 treatment increased MrgprF and CC3 staining, while it alone or synergizing with DDP decreased Ki67 and Akt phosphorylation positive staining signals, compared to reciprocal control group (Fig. 5o, p). As expected, the lung metastasis mouse model validated that the metastatic ability of B16 cells was repressed by AMG 706, as evidenced by the increased E-cadherin but decreased N-cadherin IHC staining signals, without affecting vital organs (Fig. 6a–f). These results indicated that AMG 706 functions as an agonist of MrgprF, which could be used as a novel chemotherapy drug for CM treatment in the future.

## Discussion

The incidence and mortality rate of cutaneous melanoma (CM) varies between countries, mainly dependent on racial skin phenotype and different intensities of sun exposure.^8^ Although considerable heterogeneity in melanoma has been identified by large scale genomic profiling, potential novel biomarkers that may provide new insight into the prognostication of melanoma are still in urgent demand. Our group has recently developed multiple comprehensive integrative bioinformatics methodologies to identify new biomarkers involved in hypoxic solid tumor progression.^15,40–42^ Among the candidate tumor related new biomarkers, MrgprF, a MAS related GPR family member, was revealed to be downregulated in CM due to the hypermethylation in its promoter region, which could be reversed by 5-Azacytidine treatment, a well-known anti-cancer drug.^43,44^ Consistently, 5-Azacytidine has previously been shown to behave as an anti-neoplastic agent for B16 melanoma.^45,46^

GPCRs are membrane embedded receptors, regulating pivotal biological and pathological functions, which have emerged as valuable anti-cancer drug targets,^47^ and a myriad of mutations in multiple GPCRs, including melanocortin type 1 receptor and Wnt/frizzled receptor, have been demonstrated to be involved in melanoma progression.^48^ In this study, we provided evidence showing that MrgprF overexpression inhibited, while its knockdown promoted, tumor cell proliferation and migration in vitro, and xenograft tumor growth and metastasis in vivo, respectively. Importantly, MrgprF depletion promoted the transformation and xenograft tumor formation abilities of immortalized human keratinocyte-HaCaT cells. These findings supported the hypothesis that MrgprF acts as tumor suppressor in CM (Fig. 6g). Documented findings have shown that the PI3K/Akt signaling pathway is frequently activated in CM, and our results showed that the absence of MrgprF in CM leads to constitutive activation of PI3K/Akt signaling.^12^ Consistent with the former finding that p101 bound to p110γ_1-169_ to form PI3K complex,^49^ we showed that MrgprF bound to p110γ_1-216_, suggesting that the tumor suppressor MrgprF competes with p101 to interact with the same N-terminal domain of p110γ, leading to the blockage of Class 1B PI3K complex formation and reduced signaling activation, which can be reversed by SC79 or exacerbated by AMG 706 (Fig. 6g). PI3K/Akt signaling is activated by growth factor and hormone receptors, leading to the increased cell proliferation, survival, motility, while decreased cellular apoptosis.^50^ PI3Ks have been mainly classified into two protein complexes, including Class IA and IB. The Class IA complex consists of one regulatory subunit (p85α/β, p55α/γ, and p50γ) and a catalytic subunit (p110α/β/δ), while the class IB complex contains one regulatory subunit (p101 or p87) and one catalytic subunit (p110γ).^51^ The Class I enzymes have been identified to be the main factory generating PI[3,4,5]P3 in cells. PI3Ks must interact with cellular membranes to reach their reciprocal substrate, whose function could be regulated by multiple other membrane located proteins, especially by GPCRs. How MrgprF preferentially binds to and inhibits Class IB PI3K complex is not clear, the crystal structure, protein binding components, gene interacting network, and the in vitro catalytic studies will help us decipher the underlying mechanism in the future. Collectively, our findings identify MrgprF as a new tumor suppressor in CM.

To explore the clinical value of tumor suppressor MrgprF, we discovered that AMG 706, the originally ATP-competitive inhibitor of VEGFR1, VEGFR2, VEGFR3, platelet-derived growth factor (PDGF) receptor (PDGFR) and stem cell factor (SCF) receptor (Kit), well-known receptors critical for promoting cancer cell survival and growth,^39^ increased MrgprF expression via reducing DNMT3A and DNMT3B expression, and the detail regulatory mechanism should be further characterized in the future (Fig. 6g). However, we still could not rule out the possibility that AMG 706 represses CM tumor growth via multiple signaling pathways, including VEGF related angiogenenesis and deregulated PDGFR or Kit related signaling pathways. In addition, MrgprF could form a complex with VEGFRs, PDGFR, SCF or/and Kit, and then be regulated by AMG 706. Therefore, screening new leading compound(s) specifically targeting and activating MrgprF, will benefit CM clinical treatments in the future.

## Materials and methods

### Cell culture

HEK-293T and B16 cell lines were purchased from ATCC (Manassas, VA, USA), cultured in DMEM medium supplemented with 10% Fetal Bovine Serum (Gibco) and 1% penicillin/streptomycin (Gibco). The human melanoma cell lines (A375, A875, SK-MEL-5, and SK-MEL-1) and human immortalized keratinocytes (HaCaT) were purchased from the cell bank of Kunming Institute of Zoology. Melanoma cancerous cells were cultured in DMEM/F-12 medium, while HaCaT was cultured in DMEM supplemented with 10% Fetal Bovine Serum (Gibco) and 1% penicillin/streptomycin. All cells were grown at 37 °C and 5% CO_2_.

### Constructs, lenti-viral preparation, and establishment of different cell lines

Human MrgprF full length cDNA was synthesized (Shanghai Generay Biotech) and sub-cloned into pCDH-CMV-E2F-eGFP lenti-viral vector. For shRNA knockdown experiments, independent shRNAs targeting different region of *MRGPRF* mRNA were constructed using a pLKO.1 vector (Addgene), and the oligo sequences were provided in Supplementary Table S8. Lenti-viruses were generated according to the manufacture protocol as previously documented,^42^ and indicated cells were infected by viruses twice with 48 h and 72 h viral supernatants containing 4 μg/mL polybrene. Stable cell lines were established by appropriate puromycin selection.

### Cell proliferation, BrdU incorporation, colony formation assays

Indicated tumor cells were plated onto 12-well plates, the cell numbers were subsequently counted each day using an automatic cell analyzer countstar (Shanghai Ruiyu Biotech Co.). For BrdU incorporation assay, indicated cells were cultured in 8-well chamber slides for 24 h, pretreated with or without SC79 (Beyotime, SF2730) for another 24 h, and then treated with 10 µM BrdU (Abcam, ab142567) for 20 min. Subsequently, indicated cells were fixed with 4% paraformaldehyde (PFA) at room temperature for 20 min, then incubated with BrdU primary antibody (Abcam, ab6326) followed by secondary antibody detection. The cell nuclei were stained with DAPI as previously documented.^52^ For colony formation assay, a total of 2 × 10^3^ indicated cells/well were plated onto 6-well plates treated with or without SC79, and cultured for 2 weeks at 37 °C. The medium was changed every 3 days. And 2 weeks later, indicated cells were fixed with 4% PFA for 30 min at room temperature and subsequently stained with 0.1% crystal violet for 30 min at room temperature. To examine the toxicity of AMG 706 treatment, HaCaT, A375 and A875 cells were plated into 96-well plates with 1 × 10^4^ cells per well and cultured overnight. Indicated cells were incubated with different concentrations of AMG 706 (Selleck, S5793). After drug treatment, cell viabilities were examined by sulforhodamine B (SRB) staining followed by detection at OD_515_ nm.

### Cell migration and flow cytometry assays

The wound healing assay was performed as previously documented.^42^ Briefly, indicated cells were seeded into 6-well plates (2 × 10^6^/cell) and incubated for 1 day, and then a straight line was scraped with pipette tips. Detached cells were removed. Photographs were taken at indicated time, and the relative traveled distance was measured. For the trans-well migration assay, 2 × 10^4^ cells/well in 100 μL serum free medium were plated in 24-well plate chamber insert, and the lower chamber was filled with 10% FBS. After incubation for 24 h, cells were fixed with 4% PFA, washed and then stained with 0.5% crystal violet for the capture of pictures. SC79 and AMG 706 were added into the medium of the lower chamber, respectively. For flow cytometry experiment, 4 × 10^5^ cells/well were plated onto 6-well plates, with three replicate wells in each group. After 24 h, the primary medium was changed to serum free medium and the cells were starved overnight. The next day, the serum free medium was changed to complete medium. Indicated cells were harvested and pelleted after washing with pre-chilled PBS by centrifuging, then indicated cells were re-suspended in pre-chilled 70% cold ethanol and incubated overnight at 4 °C. Finally, the cells were washed with PBS and incubated with PI at 37 °C in the dark for 30 min. To detecting the apoptotic cell population, indicated cells were treated by DDP or AMG 706, and indicated cells were then harvested and stained with an Annexin V-FITC (APC)/PI apoptosis kit (BD, 556547) according to the manufacturer’s instructions. Cell populations were determined by a flow cytometer.

### 5-Azacytidine (5-Aza) treatment and methylation specific PCR (MSP)

Indicated cells were plated in 10 cm culture dishes and incubated overnight, and then treated with 5 µM 5-Aza (Selleck, S1782) for 1 day. Indicated cells were collected for RNA and protein extraction for further Real-time RT-PCR and immunoblot experiments. For MSP assay, HaCaT and melanoma cells treated with 5-Aza or DMSO were harvested for DNA isolation using a genomic DNA kit according to the manufacturer’s instruction (Axygen, AP-MN-MS-GDNA), and then the DNA samples were treated using methylation-specific PCR (MSP) Kit (Tiangen, DP215-02). The primers used in this study are described in Supplementary Table S8. The MSP products were resolved on 2% agarose gels and visualized by ethidium bromide staining. For DNA methylation sequencing, bisulfite sequencing PCR (BSP) sequencing was used. Briefly, the genomic DNA of HaCaT and A375 cells was extracted and subjected to bisulfite conversion by EZ DNA Methylation-Gold Kit (ZYMO Research, USA). CpG islands of MrgprF in indicated cells were amplified by PCR, and then cloned into pMD19-T vector followed by transformation and amplification. Every 10 positive recombinant clones were picked for sequencing.

### Immunoprecipitation, immunoblot and immunofluorenscence

To detect the protein-protein interaction using a co-immunoprecipitation assay, exogenous different tagged forms of MrgprF, p85, p110γ or/and p101 were sub-cloned into pCDNA3.1+ vector. Constructs were transfected into HEK-293T cells and the cell lysates were subjected to immunoprecipitation with the indicated antibodies (Flag, Myc, and HA). The precipitated proteins were detected with the indicated antibodies by immunoblot. Total proteins of indicated cells were extracted using RIPA lysis buffer (Beyotime, P0013K) containing EDTA-free Protease inhibitor cocktail (Roche, 4693132001) and Phosphatase Inhibitors (Roche, 4906837001). Proteins were separated by SDS-PAGE, and were subsequently transferred onto PVDF membranes and blocked in 5% skim milk with TBS containing 0.1% Tween-20 (TBST) for 1 h at room temperature. Immunoblot was performed using the indicated primary and secondary antibodies (Supplementary Table S8). To evaluate the morphology change, indicated cells were plated onto 8-well plates and cultured for 24 h and fixed with 4% PFA. After permeabilization with 0.1% PBS + Triton X-100 for 10 min and blocking in 10% normal goat serum for 1 h at room temperature, cells were incubated with phalloidin (Sigma, P2141). Cell nuclei were stained with DAPI.

### PIP3 detection

Cell pellets were re-suspended in 150–200 μL PBS, frozen and thawed three times to break the cells. The total cell extracts were centrifuged at 2000 rpm for 10 min, and the supernatants used for PIP3 detection using Human PIP3 ELISA Kit following the manufacturer’s instruction (RUIXIN BIOTECH, RX105304H).

### Hematoxylin and eosin staining (H&E) and Immunohistochemistry (IHC)

Xenograft tumors or lung metastastic tumors were collected and fixed with 4% formaldehyde overnight. Tissues were embedded using paraffin and sectioned serially. The slides were stained with H&E for pathological analysis following a previous protocol.^42^ Immunohistochemistry was performed as described previously.^53^ Briefly, sections were deparaffinized by xylene, rehydrated with gradient ethanol, and subjected to antigen retrieval. After H_2_O_2_ treatment and blockage with 10% normal goat serum, the slides were incubated with the indicated antibodies (Supplementary Table S8) followed by an incubation with a biotinylated secondary antibody and streptavidin-HRP (Dako, K5007). All the images were captured by a binocular Nikon microscope (ECLIPSE) in High Power Field (HPF). To explore the clinical significance of MrgprF, a CM tissue microarray (TMA) containing 79 cases was obtained from Liao Ding Biotechnology Co. Ltd, Shanghai (MEL15801). MrgprF expression was scored as described previously.^53^

### Animal experiments

All animals were kept in a SPF environment and the protocols were pre-approved and conducted under the policy of Animal care and Use Committee at the Kunming Institute of Zoology, CAS. For xenograft tumor formation assay, 10 male nude mice at 4 weeks of age were divided into two groups and were injected subcutaneously with the indicated cell lines (1 × 10^6^ cells/point). Tumors were measured with a sliding caliper every 4 days post-injection and tumor volumes were calculated by the formula length × (width)^2^/2. All mice were sacrificed at the end of the experiment and tumors were collected and weighed. Each appropriate tumor tissue was fixed with formalin solution and embedded in paraffin for IHC analysis. For HaCaT xenograft tumor formation assay, 2 × 10^7^ cells/point injection was applied. After injection, tumor-free percentage was recorded. For the drug treatment in vivo assay, indicated cells were injected subcutaneously and the mice were injected with AMG 706 (7.5 mg/kg, once per day) or DDP (7 mg/kg, once per week) by intraperitoneal injection when the xenograft tumors reached to 100 mm^3^ of volume. For the lung metastasis assay, B16 cells (1 × 10^5^ cells/mouse) were injected into the tail veins of 6~8 weeks old C57BL/6 mice.^52^ After 18 days, the mice were sacrificed, and metastatic lung tumors were counted and indicated images were captured. For the AMG 706 treatment group, AMG 706 (7.5 mg/kg, once per day) was intraperitoneally injected.

### GST pull-down assay

The DNA fragments containing MrgprF_274-343_ and p110γ_1-216_ were cloned into the pGEX-4T-1 with BamH I and Not I restriction sites, and the GST fusion proteins were expressed, purified and eluted (elution buffer: 50 mM Tris pH8.8, 1 mM EDTA, 5% glycerol, 0.01 Triton X100, 50 mM NaCl, 5 mM DTT, 20 mM Glutamin) in E. coli BL_21_ (DE3). The eluted proteins were stored for competition binding assay. For GST pull-down assay, GST, GST-MrgprF_274-343_, or GST- p110γ_1-216_ proteins bound to glutathione resin, were incubated with indicated total cell extracts at 4 °C, overnight. The resin bound proteins were washed, eluted and loaded to SDS-PAGE and detected by immunoblot. GST-MrgprF_274-343_ was used for GST-pull down assay because GST-MrgprF_295-343_ could not be efficiently expressed and purified.

### Bioinformatics assay and statistical analysis

All databases used in this study are available to the public and details are provided in Supplementary Table S7. The gene expressions were tested using web-source available dataset from Gene Expression Omnibus (GEO) website,^54^ TCGA dataset, GEPIA website,^27^ GTEx Portal^55^ and CCLE dataset,^28^ respectively. For the experiment identifying MrgprF as one of the DEGs in CM, the datasets collected from articles we previously published^15^ and GEO website analyzed by online GEO2R of NCBI. To analyze the methylation status of *MRGPRF* promoter, we collected data from GSCA,^30^ DNMIVD,^31^ and MethSurv^32^ datasets. Mutation data were obtained from the GSCA and cBioportal database, and KEGG pathway enrichment analysis was performed using the GSEA software.^35^ Student’s *t*-test and one-way ANOVA test was applied using GraphPad Prism 5.0. *P < 0.05, **P < 0.01, and ***P < 0.001 were significant, ns No significance.

## Supplementary information

SUPPLEMENTAL MATERIAL

Dataset 1

Dataset 2

## Data Availability

The datasets used and/or analyzed during the current study are available from the corresponding authors on reasonable request.
