# Supplementary material for: MrgprF acts as a tumor suppressor in cutaneous melanoma by restraining PI3K/Akt signaling
Source: Signal Transduct Target Ther. 2022 May 4;7:147. doi: 10.1038/s41392-022-00945-9 (PMC9065076; doi:10.1038/s41392-022-00945-9)
Supplement: Supplementary file 1 — SUPPLEMENTAL MATERIAL [file 41392_2022_945_MOESM1_ESM.doc]

**Supplementary Materials for**

**MrgprF acts as a tumor suppressor in cutaneous melanoma by restraining PI3K/Akt signaling**

Qiushuo Shen, Yanfei Han, Kai Wu, Yaomei He, Xiulin Jiang, Peishen Liu, Cuifeng Xia, Qiuxia Xiong, Rui Liu, Qianming Chen, Yong Zhang, Song Zhao, Cuiping Yang, Yongbin Chen

Correspondence to: Song Zhao: [zhaosong@zzu.edu.cn](mailto:zhaosong@zzu.edu.cn); Cuiping Yang: cuipingyang@sjtu.edu.cn; Yongbin Chen: [ybchen@mail.kiz.ac.cn](mailto:ybchen@mail.kiz.ac.cn)

This PDF file includes:

Figures. S1 to S5

Tables S1 to S9


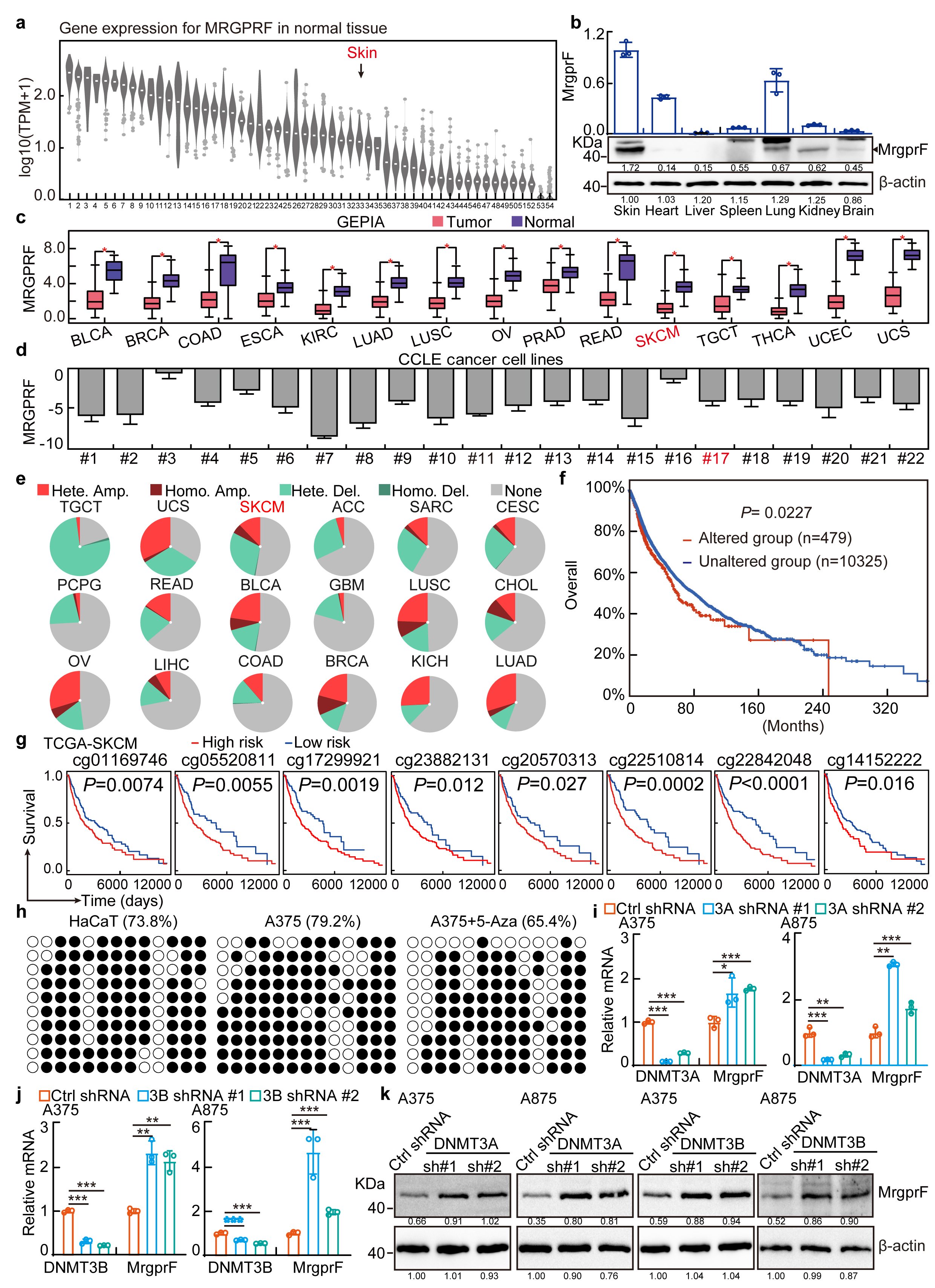


**Figure. S1. MrgprF was downregulated in pan-cancer. (a)** The relative mRNA expression pattern of MrgprF in various normal human tissues using GTEX database analysis. The tissue names corresponding to each number were indicated in Table S2. **(b)** The relative mRNA (top) and protein (bottom) expressions of MrgprF in vital organs of mouse. Black arrow indicating MrgprF. **(c)** Expression patterns of MrgprF in pan-cancers examined by GEPIA. Full names for the indicated cancer types are shown in Table S3. **(d)** The relative mRNA expression pattern of MrgprF in various cancerous cell lines as examined by the CCLE dataset. The cancer types corresponding to each number were indicated in Table S4. **(e)** The mutation status of MrgprF in pan-cancer examined by the GSCALite dataset. Hete: Heterozygous; Homo: Homozygous; Amp: Amplification; Dele: Deletion. Details were indicated in Tables S3 and S5. **(f)** Tumor patients with genetic alteration in *MRGPRF* exhibit a worse overall survival rate as examined by cBioPortal. **(g)** The prognostic value of *MRGPRF* promoter methylation as examined by the Kaplan-Meier Plotter using the MethSurv database. **(h)** Methylation status of the MrgprF promoter by DNA methylation sequencing, with or without 5-Azacytidine (5-Aza) treatment. **(i-k)** Knockdown of DNMT3A (i) or DNMT3B (j), respectively, promoted MrgprF expression examined by Real-time RT-PCR and immunoblot (k). 3A= DNMT3A , 3B= DNMT3B. Quantified results for all the immunoblots are indicated below, which are normalized to the β-actin signal, compared to reciprocal control. Bars are the mean value ± SD. * *P* < 0.05, ** *P* < 0.01, *** *P* < 0.001.

**
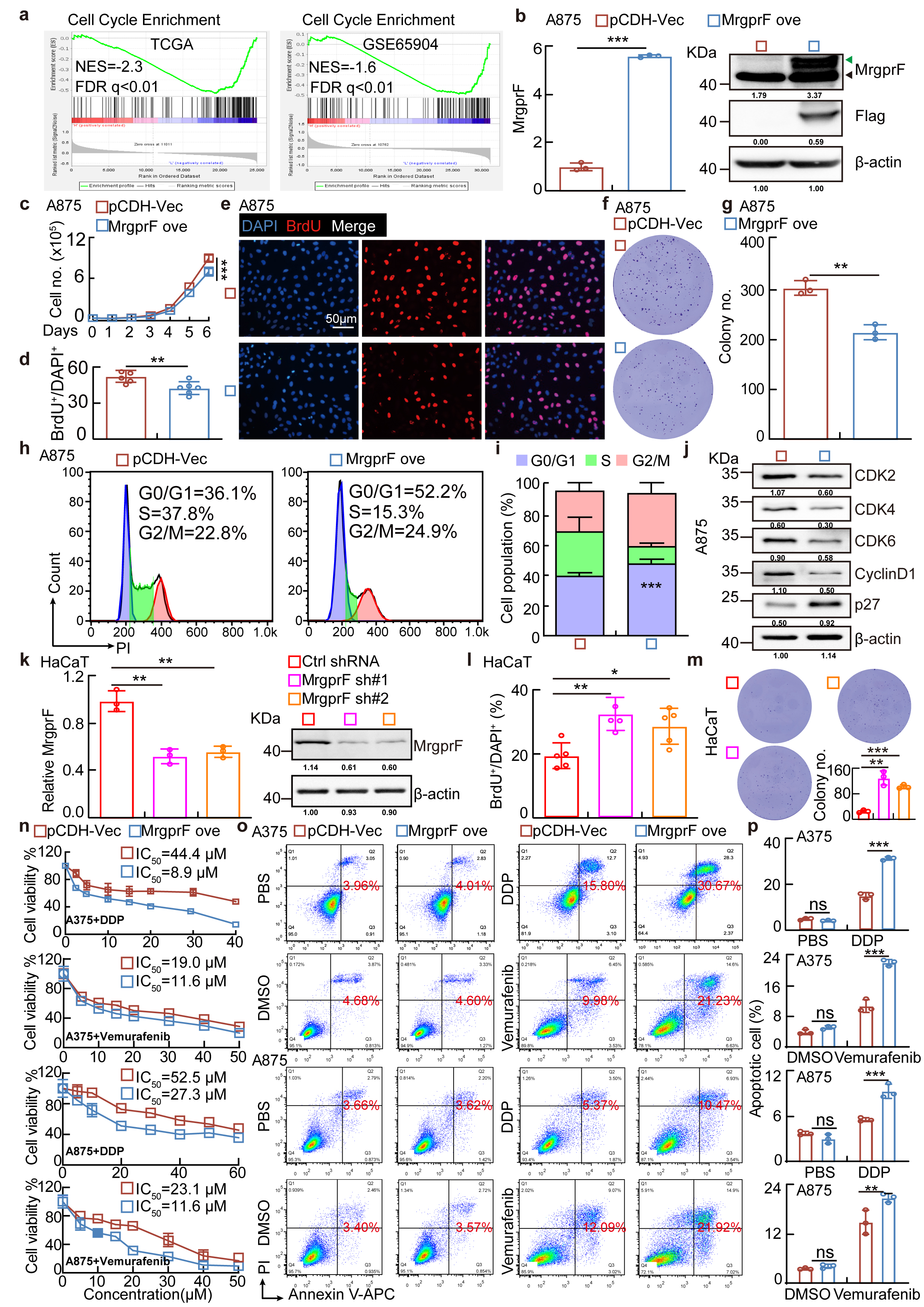
**

**Figure. S2. MrgprF inhibits tumor cell growth. (a)** The cell cycle signaling pathways were enriched with GSEA analysis using the TCGA and GSE65904 datasets. **(b)** Establishment of MrgprF overexpression in A875 cells, verified by Real-time RT-PCR (left) and immunoblot (right). Green arrow: exogenous MrgprF-Flag; black arrow: endogenous MrgprF. **(c)** Indicated cell growth was examined by daily counting. **(d-e)** MrgprF overexpression reduced BrdU positive staining cells as determined by the BrdU incorporation assay in A875 cells. (d) Quantification data for (e). Scale bar=50μm. **(f-g)** MrgprF overexpression reduced the colony formation ability of A875 cells. (g) Quantification data for (f). **(h-i)** MrgprF overexpression increased G0/G1 phase arrested cell population as examined by FACS analysis. (i) Quantification data for (h). **(j)** Overexpression of MrgprF decreased CDK2, CDK4, CDK6 and Cyclin D1 expressions, but increased p27 expression. **(k)** Establishment of MrgprF knockdown cell lines in HaCaT cells, verified by Real-time RT-PCR (left) and immunoblot (right), respectively. **(l)** BrdU incorporation assays were performed in the indicated cells. **(m)** MrgprF knockdown promoted colony formation abilities of HaCaT cells. Quantification data is shown. **(n-p)** Cellular apoptosis with or without DDP or BRAF inhibitor (Vemurafenib) treatment upon MrgprF overexprssion was examined by cell viability assay (n) and FACS analysis (o) in A375 and A875 cells. (p) Quantification data for (o). Quantified results for all the immunoblots are indicated below, which are normalized to the β-actin signal, compared to reciprocal control. Bars are the mean value ± SD. * *P* < 0.05, ** *P* < 0.01, *** *P* < 0.001.

**
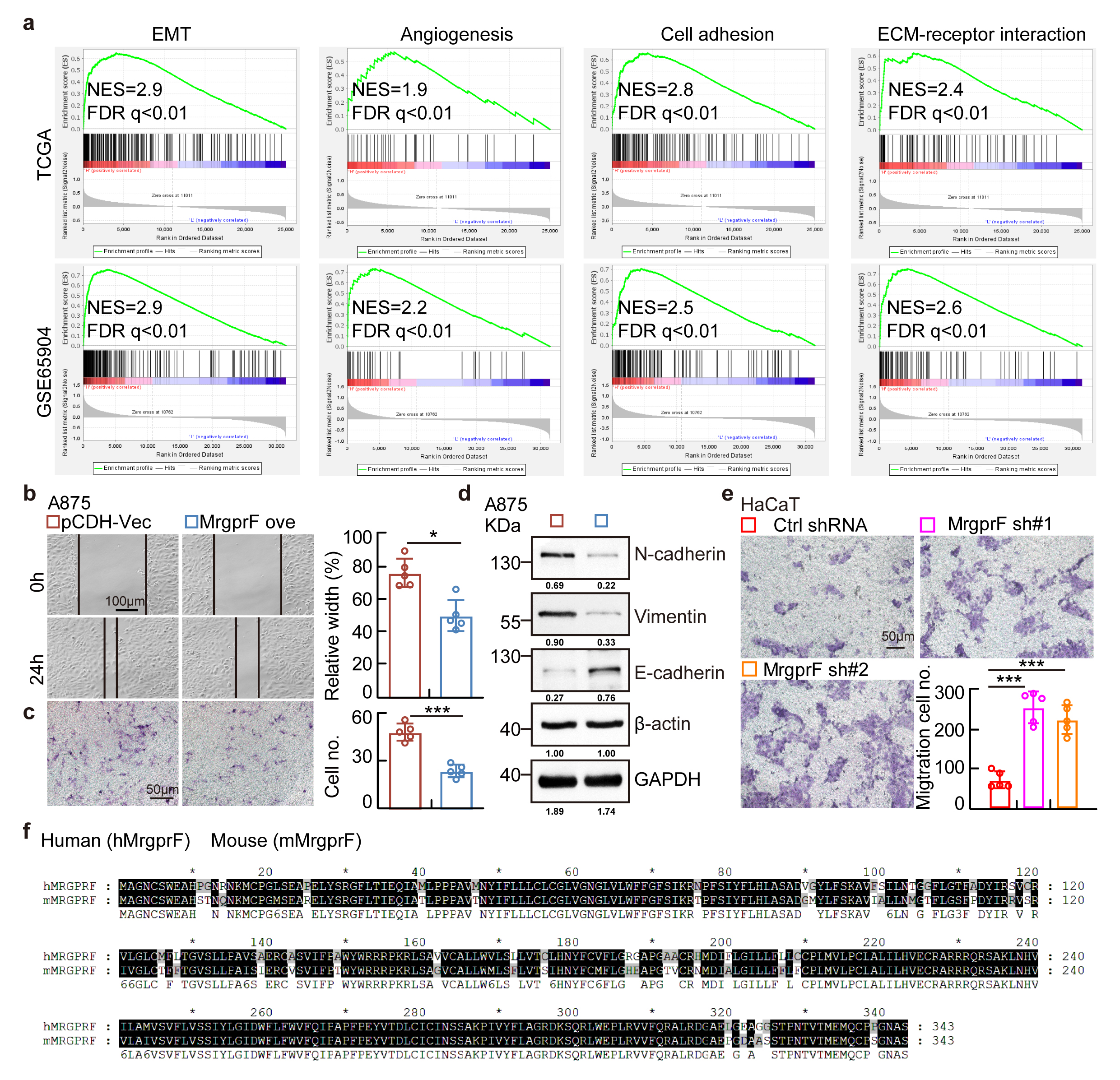
**

**Figure. S3. Forced expression of MrgprF inhibited melanoma cell migration. (a)** Cell migration related signaling pathways, including epithelial-mesenchymal transition (EMT), angiogenesis, cell adhesion and ECM-receptor interaction, were enriched by GSEA analysis using TCGA and GSE65904 datasets. **(b-c)** Wound healing (b) and trans-well assays (c) were performed to examine A875 cell migration ability upon MrgprF overexpression. Quantification data is shown in the right panel. **(d)** The protein expressions of E-cadherin, N-cadherin and Vimentin were detected by immunoblot with the indicated antibodies in A875 cell extracts. **(e)** Depletion of MrgprF promoted HaCaT cell migration by trans-well assay. Quantification results are also shown. Scale bar=50μm. **(f)** Protein sequence alignment between human and mouse MrgprF. Quantified results for all the immunoblots are indicated below, which are normalized to the β-actin signal, compared to reciprocal control. Bars are the mean value ± SD. * *P* < 0.05, ** *P* < 0.01, *** *P* < 0.001.

**
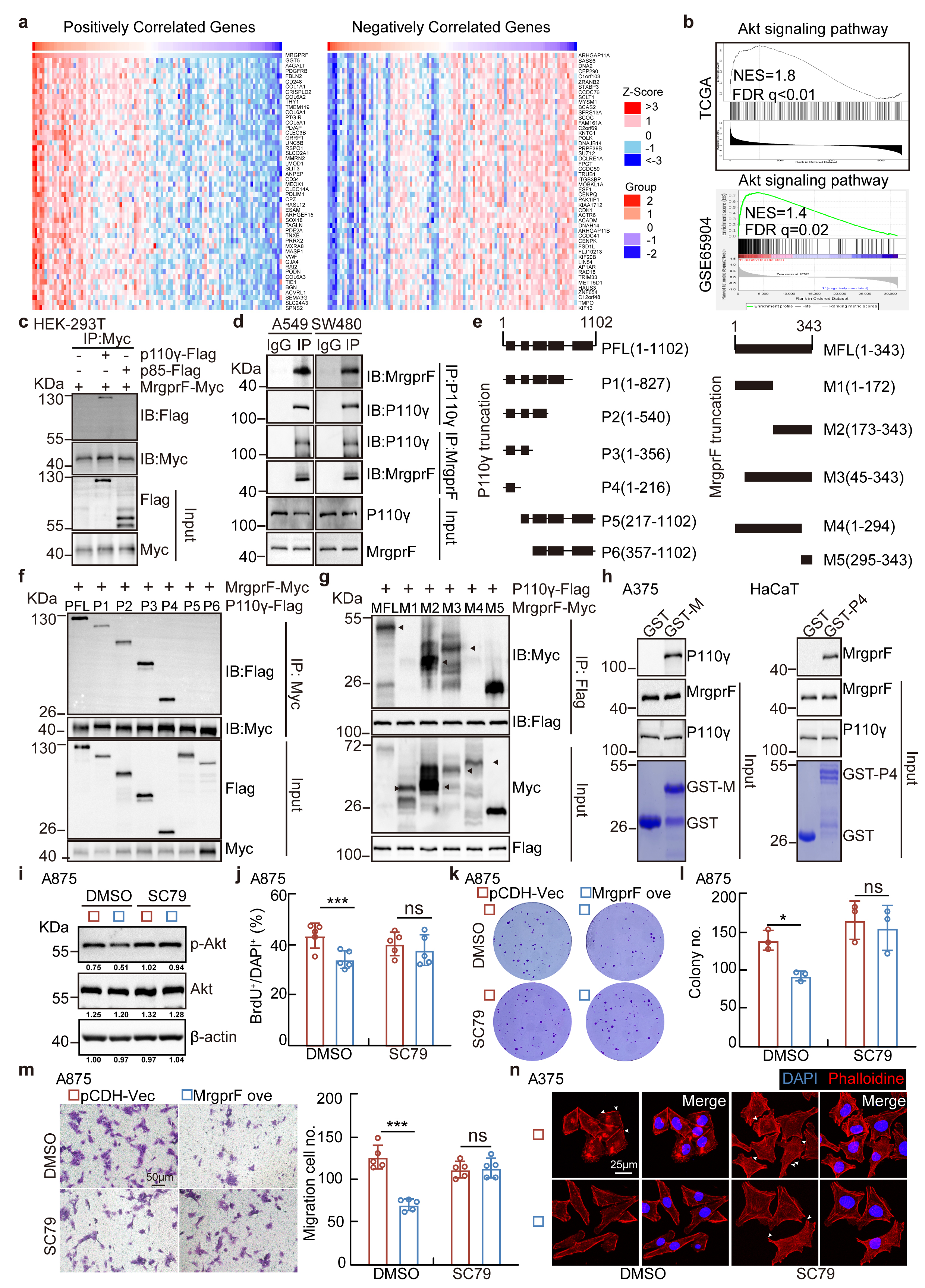
**

**Figure. S4. MrgprF reduced PI3K/Akt signaling in melanoma. (a)** The heat maps showing top 50 genes positively and negatively correlated with MrgprF in CM using the LinkedOmics portal dataset. **(b)** GSEA analysis revealed that MrgprF is involved in Akt signaling in CM as examined by the TCGA and GSE65094 datasets, respectively. **(c)** Co-immunoprecipitation assay (co-IP) showing that MrgprF interacted with p110γ, but not p85. **(d)** Co-IP assay showing that endogenous MrgprF interacted with p110γ in non-small cell lung cancer cell line A549 and colorectal cancer cell line SW480. **(e)** The schematic picture of the mutation constructs for p110γ (left) and MrgprF (right). PFL=p110γ full length; MFL=MrgprF full length. **(f-g)** Co-IP assay mapping the domain mediating MrgprF and p110γ interaction. Black arrows pointing to the indicated proteins. **(h)** GST pull-down assay was performed to determine the physical interaction between MrgprF and p110γ in indicated cells. GST-M=GST-MrgprF_274-343_, GST-P4=GST-p110γ_1-216_. GST-MrgprF_274-343_ was used for GST-pull down assay because GST-MrgprF_295-343_ could not be efficiently expressed and purified. **(i)** Indicated cells stably expressing empty vector or MrgprF in A875 were treated with DMSO or SC79 (15 μM). Indicated cell lysates were examined by immunoblot with the indicated antibodies. **(j)** The quantification data for the BrdU incorporation assay in A875 upon MrgprF oeverexpression treated with DMSO or SC79 (15 μM). **(k-l)** The colony formation ability of A875 after MrgprF forced expression treated with DMSO or SC79 (15 μM). (l) Quantification data for (k). **(m)** The trans-well assay for A875 upon MrgprF overexpression treated with DMSO or SC79 (15 μM). Quantification data was indicated (right). Scale bar=50 μm. **(n)** Immunofluorescence staining of Phalloidine in A375 after MrgprF overexpression treated with DMSO or SC79 (15 μM). White arrows pointing to the pseudopodia like structure. Scale bar=25μm. Quantified results for all the immunoblots are indicated below, which are normalized to the β-actin signal, compared to reciprocal control. Bars are the mean value ± SD. * *P* < 0.05, ** *P* < 0.01, *** *P* < 0.001.

**
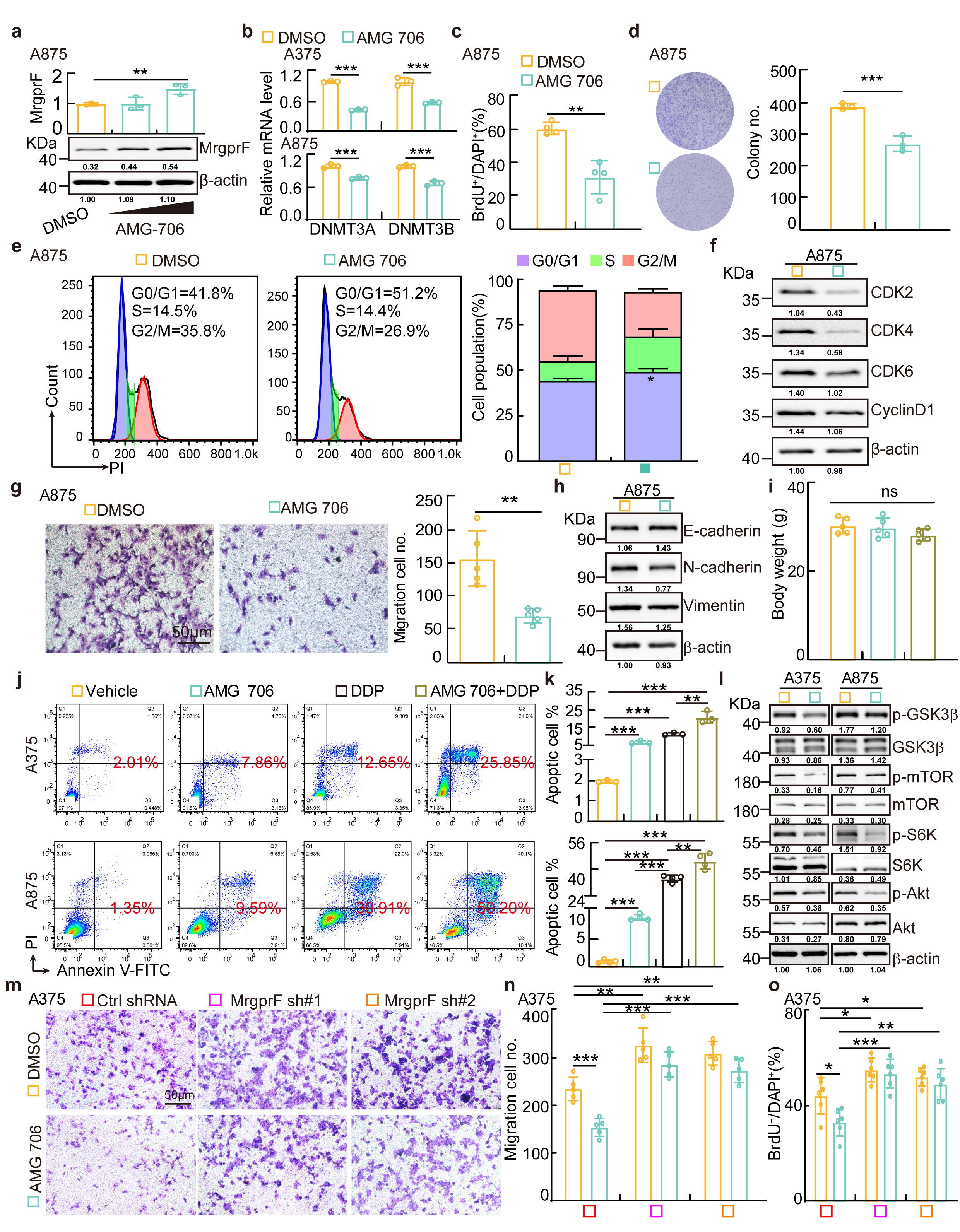
**

**Figure. S5. AMG 706 is a potent MrgprF activator. (a)** AMG 706 (5 and 10 μM) 24 hours treatment increased MrgprF expressions as examined by Real-time RT-PCR (left) and immunoblot (right) in A875 cells. **(b)** AMG 706 treatment reduced DNMT3A and DNMT3B expressions in tumor cells, compared to DMSO control treatment group. **(c-d)** AMG 706 (10 μM) treatment in A875 cells decreased cell proliferation as examined by BrdU incorporation (c) and colony formation (d) assays. Quantification data is shown. **(e-f)** AMG 706 (10 μM) treatment in A875 cells promoted the G0/G1 phase arrested cell population examined by FACS (e) and immunoblot (f). Indicated cell lysates were probed with indicated antibodies. Quantification data is shown. **(g-h)** AMG 706 (10 μM) treatment in A875 cells suppressed cell migration as examined by trans-well (g) and immunoblot (h). Indicated cell lysates were probed with indicated antibodies. Quantification data is shown. Scale bar=50μm. **(i)** Xenograft mouse body weights with DDP (7mg/kg) or/and AMG 706 (7.5 mg/kg) treatments are presented. **(j-k)** DDP (15 μM) treatment induced cellular apoptosis which was increased by AMG 706 (15 μM) treatment, as examined by FACS analysis in A375 and A875 cells. (j) Quantification data for (i). **(l)** AMG 706 treatment decreased PI3K/Akt signaling activity as examined by immunoblot with the indicated antibodies in A375 and A875 cells. **(m-o)** MrgprF knockdown markedly reduced AMG 706 treatment effects as examined by trans-well (m-n) and BrdU incorporation assays (o). Quantified results for all the immunoblots are indicated below, which are normalized to the β-actin signal, compared to reciprocal control. Bars are the mean value ± SD. * *P* < 0.05, ** *P* < 0.01, *** *P* < 0.001.

**Supplementary Tables**

**Table S1. The candidate genes were identified by integrative analyses using web-source available datasets (see attached Microsoft Excel file).**

**Table S2. Denotation of numbers in Figure. S1a**

| Number | Keywords | Number | Keywords |
| --- | --- | --- | --- |
| 1 | Uterus | 28 | Spleen |
| 2 | Colon - Sigmoid | 29 | Esophagus - Mucosa |
| 3 | Cervix - Ectocervix | 30 | Skin - Not Sun Exposed (Suprapubic) |
| 4 | Fallopian Tube | 31 | Testis |
| 5 | Esophagus - Gastroesophageal Junction | 32 | Minor Salivary Gland |
| 6 | Cervix - Endocervix | 33 | Kidney - Cortex |
| 7 | Esophagus - Muscularis | 34 | Heart -Atrial Appendage |
| 8 | Artery - Tibial | 35 | Kidney -Medulla |
| 9 | Artery - Aorta | 36 | Pituitary |
| 10 | Bladder | 37 | Adrenal Gland |
| 11 | Vagina | 38 | Heart - Left Ventricle |
| 12 | Artery - Coronary | 39 | Liver |
| 13 | Colon - Transverse | 40 | Muscle - Skeletal |
| 14 | Prostate | 41 | Brain - Cortex |
| 15 | Adipose - Subcutaneous | 42 | Pancreas |
| 16 | Adipose - Visceral (Omentum) | 43 | Brain - Spinal cord (cervical c-1) |
| 17 | Nerve - Tibial | 44 | Brain - Frontal Cortex(BA9) |
| 18 | Ovary | 45 | Brain - Hypothalamus |
| 19 | Breast - Mammary Tissue | 46 | Brain - Amygdala |
| 20 | Cells - Cultured fibroblasts | 47 | Brain - Anterior cingulate cortex (BA24) |
| 21 | Lung | 48 | Brain - Substantia nigra |
| 22 | SmallIntestine - Terminal Ileum | 49 | Brain - Hippocampus |
| 23 | Brain - Cerebellum | 50 | Brain - Putamen (basalganglia) |
| 24 | Brain - Cerebellar Hemisphere | 51 | Brain - Caudate (basal ganglia) |
| 25 | Skin - Sun Exposed (Lower leg) | 52 | Brain - Nucleus accumbens (basal ganglia) |
| 26 | Stomach | 53 | Cells - EBV-transformed lymphocytes |
| 27 | Thyroid | 54 | Whole Blood |

**Table S3.** **The full names of cancer types shown in this study.**

| **Cancer Type** | **Full Name** |
| --- | --- |
| BLCA | Bladder Urothelial Carcinoma |
| BRCA | Breast invasive carcinoma |
| CESC | Cervical squamous cell carcinoma and endocervical adenocarcinoma |
| COAD | Colon adenocarcinoma |
| ESCA | Esophageal carcinoma |
| HNSC | Head and Neck squamous cell carcinoma |
| KIRC | Kidney renal clear cell carcinoma |
| KIRP | Kidney renal papillary cell carcinoma |
| LIHC | Liver hepatocellular carcinoma |
| LUAD | Lung adenocarcinoma |
| LUSC | Lung squamous cell carcinoma |
| OV | Ovarian serous cystadenocarcinoma |
| PAAD | Pancreatic adenocarcinoma |
| PRAD | Prostate adenocarcinoma |
| READ | Rectum adenocarcinoma |
| SARC | Sarcoma |
| SKCM | Skin Cutaneous Melanoma |
| TGCT | Testicular Germ Cell Tumors |
| THCA | Thyroid carcinoma |
| THYM | Thymoma |
| UCEC | Uterine Corpus Endometrial Carcinoma |
| UCS | Uterine Carcinosarcoma |

**Table S4. Denotation of numbers in Figure. S1c**

| Number | Keywords | Number | Keywords |
| --- | --- | --- | --- |
| #1 | Autonomic ganglia | #12 | Oesophagus |
| #2 | Biliary tract | #13 | Ovary |
| #3 | Bone | #14 | Pancreas |
| #4 | Breast | #15 | Prostate |
| #5 | Central nervous | #16 | Salivary |
| #6 | Endometrium | #17 | Skin melanoma |
| #7 | Hematopoietic | #18 | Soft tissue |
| #8 | Kidney | #19 | Stomach |
| #9 | Large intestine | #20 | Thyroid |
| #10 | Liver | #21 | Upper tract aerodigestive |
| #11 | Lung | #22 | Urinary |

**Table S5. Mutation pattern of *MRGPRF*** in pan-cancer.

| **Cancer type** | **symbol** | **a_total** | **d_total** | **a_hete** | **d_hete** | **a_homo** | **d_homo** | **Entrez**  **ID** |
| --- | --- | --- | --- | --- | --- | --- | --- | --- |
| ACC | MRGPRF | 4.444444 | 27.77778 | 4.444444 | 27.77778 | 0 | 0 | 116535 |
| BLCA | MRGPRF | 29.41176 | 18.13725 | 22.54902 | 17.64706 | 6.862745 | 0.490196 | 116535 |
| BRCA | MRGPRF | 31.66667 | 13.05556 | 20.74074 | 12.96296 | 10.92593 | 0.092593 | 116535 |
| CESC | MRGPRF | 13.22034 | 25.76271 | 11.18644 | 25.42373 | 2.033898 | 0.338983 | 116535 |
| CHOL | MRGPRF | 19.44444 | 16.66667 | 11.11111 | 16.66667 | 8.333333 | 0 | 116535 |
| COAD | MRGPRF | 11.3082 | 14.41242 | 11.3082 | 13.96896 | 0 | 0.443459 | 116535 |
| DLBC | MRGPRF | 20.83333 | 4.166667 | 20.83333 | 4.166667 | 0 | 0 | 116535 |
| ESCA | MRGPRF | 51.08696 | 9.782609 | 26.08696 | 9.782609 | 25 | 0 | 116535 |
| GBM | MRGPRF | 3.119584 | 17.33102 | 2.599653 | 17.33102 | 0.519931 | 0 | 116535 |
| HNSC | MRGPRF | 38.31418 | 10.91954 | 22.60536 | 10.72797 | 15.70881 | 0.191571 | 116535 |
| KICH | MRGPRF | 25.75758 | 12.12121 | 25.75758 | 12.12121 | 0 | 0 | 116535 |
| KIRC | MRGPRF | 6.25 | 3.598485 | 6.25 | 3.598485 | 0 | 0 | 116535 |
| KIRP | MRGPRF | 3.472222 | 5.555556 | 3.125 | 5.555556 | 0.347222 | 0 | 116535 |
| LAML | MRGPRF | 3.664922 | 0 | 3.664922 | 0 | 0 | 0 | 116535 |
| LGG | MRGPRF | 10.52632 | 4.093567 | 10.33138 | 4.093567 | 0.194932 | 0 | 116535 |
| LIHC | MRGPRF | 13.51351 | 14.59459 | 8.378378 | 14.59459 | 5.135135 | 0 | 116535 |
| LUAD | MRGPRF | 33.13953 | 11.24031 | 29.84496 | 11.24031 | 3.294574 | 0 | 116535 |
| LUSC | MRGPRF | 33.33333 | 17.16567 | 24.3513 | 17.16567 | 8.982036 | 0 | 116535 |
| MESO | MRGPRF | 19.54023 | 2.298851 | 19.54023 | 2.298851 | 0 | 0 | 116535 |
| OV | MRGPRF | 35.40587 | 16.58031 | 29.8791 | 16.58031 | 5.52677 | 0 | 116535 |
| PAAD | MRGPRF | 10.86957 | 7.065217 | 9.23913 | 7.065217 | 1.630435 | 0 | 116535 |
| PCPG | MRGPRF | 3.703704 | 22.22222 | 2.469136 | 22.22222 | 1.234568 | 0 | 116535 |
| PRAD | MRGPRF | 9.552846 | 0.813008 | 7.723577 | 0.609756 | 1.829268 | 0.203252 | 116535 |
| READ | MRGPRF | 15.75758 | 20.60606 | 15.15152 | 20.60606 | 0.606061 | 0 | 116535 |
| SARC | MRGPRF | 14.00778 | 27.62646 | 11.67315 | 27.62646 | 2.33463 | 0 | 116535 |
| SKCM | MRGPRF | 17.16621 | 30.24523 | 11.9891 | 29.70027 | 5.177112 | 0.544959 | 116535 |
| STAD | MRGPRF | 24.26304 | 11.11111 | 19.27438 | 10.6576 | 4.988662 | 0.453515 | 116535 |
| TGCT | MRGPRF | 2 | 78 | 2 | 76.66667 | 0 | 1.333333 | 116535 |
| THCA | MRGPRF | 1.002004 | 1.603206 | 1.002004 | 1.603206 | 0 | 0 | 116535 |
| THYM | MRGPRF | 0.813008 | 5.691057 | 0.813008 | 5.691057 | 0 | 0 | 116535 |
| UCEC | MRGPRF | 10.76067 | 10.01855 | 8.90538 | 9.833024 | 1.855288 | 0.185529 | 116535 |
| UCS | MRGPRF | 33.92857 | 32.14286 | 32.14286 | 32.14286 | 1.785714 | 0 | 116535 |
| UVM | MRGPRF | 12.5 | 1.25 | 12.5 | 1.25 | 0 | 0 | 116535 |

**Abbreviation**: **a_total:** amplification_total; **d_total:** deletion_total; **a_hete:** amplification_heterozygous; **d_hete:** deletion_heterozygous; **a_homo:** amplification_homozygous; **d_homo:** deletion_ homozygous.

**Table S6. The pathological characteristics of patients with melanoma (n= 79) in tissue microarray.**

| **Patients characteristics** | **No. (%)** |
| --- | --- |
| **Age (years)** |  |
| ≤56 (n=40) | 50.63% |
| **>**56 (n=39) | 49.37% |
| **Gender** |  |
| Female (n=33) | 41.77% |
| Male (n=46) | 58.23% |
| **Clinical stages** |  |
| I | 21.52% |
| II | 37.97% |
| III | 24.05% |
| IV | 16.46% |
| **Lymph nodes** |  |
| N0 | 55.70% |
| N1/N2/N3 | 44.30% |
| **Survival status** |  |
| Alive | 45.57% |
| Dead | 54.43% |

**Table S7. Bioinformatics** databases used in this study.

| **Name** | **Keywords** | **Link** | **Targets** |
| --- | --- | --- | --- |
| GEO | Gene Expression Omnibus | <https://www.ncbi.nlm.nih.gov/geo/> | To examine the differentially expressed genes in melanoma, compared to normal tissue. |
| CCLE | Cancer Cell Line Encyclopedia | <https://sites.broadinstitute.org/ccle> | To examine the expression of MrgprF in different tumor cell lines. |
| GSCA | Gene Set Cancer Analysis | [http://bioinfo.life.hust.edu.cn/GSCA/#/expression](http://bioinfo.life.hust.edu.cn/GSCA/#/expression) | To examine the gene mutation pattern, methylation level and targeting drugs. |
| DNMIVD | DNA Methylation Interactive Visualization Database | <http://119.3.41.228/dnmivd/index/> | To examine the methylation status. |
| MethSurv | DNA methylation and its relation to cancer survival | <https://biit.cs.ut.ee/methsurv/> | To examine the methylation status. |
| SMART | Shiny Methylation Analysis Resource Tool | <http://www.bioinfo-zs.com/smartapp/> | To examine the methylation status. |
| GEPIA | Gene Expression Profiling Interactive Analysis | <http://gepia.cancer-pku.cn/index.html> | To examine the expression pattern of MrgprF. |
| GTEx | Genotype-Tissue Expression | [https://www.gtexportal.org](https://www.gtexportal.org/) | To examine the expression pattern of MrgprF in human normal tissues. |
| cBioportal |  | <http://www.cbioportal.org/> | To examine the mutation pattern of MrgprF in melanoma. |
| TCGAportal |  | [http://www.tcgaportal.org](http://www.tcgaportal.org/) | To examine the correlation between MrgprF and other gene in melanoma. |
| LinkedOmics |  | http://www.linkedomics.org/login.php | To explore the signaling pathways related to MrgprF in melanoma. |

**Table S8. Antibodies and oligos used in this study.**

| **Antibody Name** | **Catalog Number** | **Dilution** | **Supplier** | **Species** |
| --- | --- | --- | --- | --- |
| E-cadherin | ab40772 | 1:500 | abcam | Rabbit |
| N-cadherin | ab18203 | 1:1000 | abcam | Rabbit |
| Vimentin | 103661-1-AP | 1:1000 | Proteintech | Rabbit |
| PARP | 9542S | 1:1000 | CST | Rabbit |
| Cleaved caspase3 | 9661S | 1:500 | CST | Rabbit |
| HA | sc-7392 | 1:200 | santa cruz | Mouse |
| HA | 3724T | 1:1000 | CST | Rabbit |
| Flag | F1804 | 1:200 | Sigma | Mouse |
| Flag | 14793S | 1:1000 | CST | Rabbit |
| Myc | sc-40 | 1:200 | santa cruz | Mouse |
| Myc | 18583S | 1:1000 | CST | Rabbit |
| CDK2 | 10122-1-AP | 1:1000 | Proteintech | Rabbit |
| CDK6 | ab124821 | 1:1000 | abcam | Rabbit |
| Cyclin D1 | 60186-1-1g | 1:1000 | Proteintech | Mouse |
| p27 | 610241 | 1:1000 | BD | Mouse |
| β-actin | 60008-1-1g | 1:5000 | Proteintech | Mouse |
| Akt | 9272 | 1:1000 | CST | Rabbit |
| p-Akt | 9271 | 1:1000 | CST | Rabbit |
| GSK3β | 5676 | 1:1000 | CST | Rabbit |
| p-GSK3β | 9336 | 1:1000 | CST | Rabbit |
| S6K | 9202 | 1:1000 | CST | Rabbit |
| p-S6K | 9205 | 1:1000 | CST | Rabbit |
| mTOR | 2983 | 1:2000 | CST | Rabbit |
| p-mTOR | 5536 | 1:2000 | CST | Rabbit |
| MRGPRF | ab254756 | 1:2000 | abcam | Rabbit |
| Ki67 | Kit-0005 | 1:1000 | MXB | Mouse/Rabbit |
| BrdU | ab6326 | 1:500 | abcam | Rat |
| GST | 2624 | 1:5000 | CST | Mouse |
| Primer Name | Primer Sequences(5'-3') | | | |
| Human β-actin_F | AAGTGTGACGTGGACATCCGC | | | |
| Human β-actin_R | CCGGACTCGTCATACTCCTGCT | | | |
| Human *MRGPRF*_F | CGTCACTGACCTGTGCATCT | | | |
| Human *MRGPRF*_R | CTCCATGGTGACTGTGTTGG | | | |
| Mouse *MRGPRF*_F | ATGGCCGGAAACTGTTCATGG | | | |
| Mouse *MRGPRF*_R | TGGTCAGAAATCCTCTGCTGTA | | | |
| Mouse GAPDH_F | CTCAACTACATGGTCTACATGTTCCA | | | |
| Mouse GAPDH_R | CCATTCTCGGCCTTGACTGT | | | |
| MSP_M_ *MRGPRF*_F | TCGGAGTTATGGGAAATAAAC | | | |
| MSP_M_*MRGPRF*_R | CTTCCCGTAAACTTAATATATCCGAC | | | |
| MSP_U_*MRGPRF*_F | TTTGGAGTTATGGGAAATAAATG | | | |
| MSP_U_*MRGPRF*_R | TCCCATAAACTTAATATATCCAAC | | | |
| Ctr shRNA_F | GCACTACCAGAGCTAACTCAG | | | |
| Ctr shRNA_R | CTGAGTTAGCTCTGGTAGTGC | | | |
| Human *MRGPRF* shRNA #1_F | GGATCGACTGGTTCCTCTTCT | | | |
| Human *MRGPRF* shRNA #1_R | AGAAGAGGAACCAGTCGATCC | | | |
| Human *MRGPRF* shRNA #2_F | GCATTATCAGTGAGCAAATGT | | | |
| Human *MRGPRF* shRNA #2_R | ACATTTGCTCACTGATAATGC | | | |

**Table S9. The correlation between *MRGPRF* expression and GDSC drug sensitivity in pan-cancer (see attached Microsoft Excel file).**
